# The S phase checkpoint promotes the Smc5/6 complex dependent SUMOylation of Pol2, the catalytic subunit of DNA polymerase ε

**DOI:** 10.1371/journal.pgen.1008427

**Published:** 2019-11-25

**Authors:** Alicja Winczura, Rowin Appanah, Michael H. Tatham, Ronald T. Hay, Giacomo De Piccoli

**Affiliations:** 1 Warwick Medical School, University of Warwick, Coventry, United Kingdom; 2 Centre for Gene Regulation and Expression, School of Life Sciences, University of Dundee, United Kingdom; University of California San Francisco, UNITED STATES

## Abstract

Replication fork stalling and accumulation of single-stranded DNA trigger the S phase checkpoint, a signalling cascade that, in budding yeast, leads to the activation of the Rad53 kinase. Rad53 is essential in maintaining cell viability, but its targets of regulation are still partially unknown. Here we show that Rad53 drives the hyper-SUMOylation of Pol2, the catalytic subunit of DNA polymerase ε, principally following replication forks stalling induced by nucleotide depletion. Pol2 is the main target of SUMOylation within the replisome and its modification requires the SUMO-ligase Mms21, a subunit of the Smc5/6 complex. Moreover, the Smc5/6 complex co-purifies with Pol ε, independently of other replisome components. Finally, we map Pol2 SUMOylation to a single site within the N-terminal catalytic domain and identify a SUMO-interacting motif at the C-terminus of Pol2. These data suggest that the S phase checkpoint regulate Pol ε during replication stress through Pol2 SUMOylation and SUMO-binding ability

## Author summary

Chromosome duplication is essential for cell proliferation. Mistakes during this process introduce mutations, duplications, deletions and rearrangements of the genetic information. Cells have evolved a monitoring pathway that supervises DNA replication, called S phase checkpoint; this regulates many aspects of the cell biology in response of defects during DNA replication, so to promote the complete and faithful completion of DNA replication once the obstacles are eliminated. While many details of the S phase checkpoint are known, how this pathway regulates the machine copying the DNA is still largely poorly understood. Here we present a novel mechanism of action of the S phase checkpoint preferentially observed in response to replication forks stalling. We show that the catalytic subunit of DNA Pol ε (Pol2), is the preferential target of modification with SUMO among the replication machinery components. This mono-SUMOylation depends on the S phase checkpoint factors Rad53, Mrc1 and Ctf18. Moreover, Pol ε is bound and SUMOylated by the Smc5/6 complex. Finally, we identified the site of the Pol2 modification and a SUMO binding-motif, suggesting a possible mechanism of action for this modification.

## Introduction

The maintenance of genome stability requires the faithful and complete duplication of the chromosomes in each cell cycle. Therefore, pathways controlling the formation, activity and repair of replication forks play a key role in safeguarding cell viability and act as a bulwark against cell transformation [1, 2]. These pathways must be coordinated, integrating different stimuli within the cell, and the cross-talk between different post-translational modifications plays a critical role in their regulation. For example, the initiation of chromosome replication is positively regulated by the phosphorylation of Mcm4 by DDK (Dbf4-dependent kinase) [3–5], and negatively controlled by SUMOylation of the origin-bound double hexamer of Mcm2-7. In fact, SUMOylation promotes the recruitment of Rif1 and the Glc7 phosphatase, thus reversing DDK activity and counteracting origin firing [6–8]. The analysis of the cross-talk of different signalling pathways is therefore often necessary to understand how complex processes are fine-tuned.

Origin firing leads to the assembly of the replisome, which faithfully duplicates the entirety of the genome [9]. Eukaryotic replisomes consist of the CMG helicase (Cdc45/Mcm2-7/GINS), DNA polymerase epsilon (Pol ε)–which synthesises the leading strand at replication forks -, DNA polymerase alpha (Pol α),—which makes RNA-DNA primers for Okazaki-fragments synthesis before their handover to DNA polymerase delta (Pol ∂) during lagging strand synthesis -, and a series of other factors that structurally and functionally coordinate the activity of DNA unwinding and DNA synthesis, such as the Mrc1/Tof1/Csm3 sub-complex or the trimeric “interaction hub” Ctf4 [10–12]. The replisome then duplicates the chromosome template at rates between 1 and 2 kb min^−1^ [13, 14]. Nevertheless, many obstacles can impede the progression of replication forks, ranging from DNA-protein or DNA/RNA barriers, insults of the DNA template caused by endogenous and exogenous sources, or insufficient levels of dNTPs for processive DNA synthesis. All these obstacles ultimately halt the progression of the fork and are often collectively referred to as ‘replication stress’ [2, 15]. These challenges to replication differ in nature and require different strategies to overcome them. For example, whereas damage caused by UV light causes the build-up of single-stranded DNA (ssDNA) gaps behind the fork, treatment with hydroxyurea (HU) leads to limited ssDNA accumulation close to the replisome [16, 17]. In response to this accumulation, cells activate the S phase and DNA damage checkpoints, two overlapping but distinct pathways that recognise the accumulation of single-stranded DNA at replication forks or closely behind them, respectively [18]. In budding yeast, both pathways require the activation of the checkpoint kinase Mec1, which critically phosphorylates the mediators Mrc1 and Rad9 (most prominently for the S phase and DNA damage checkpoint, respectively). This leads to the recruitment, phosphorylation and activation of the effector kinase Rad53, which regulates several different pathways in the cell, including cell cycle control, inhibition of late origin firing, gene expression, regulation of nucleases and helicases, and control of chromatin remodelling enzymes [19, 20]. Strikingly, checkpoint kinases also phosphorylate several replisome components, such as Cdc45, Ctf4, Tof1, Dpb4, Pol 31, Pol1, several MCMs subunits and GINS [21–26]. This suggests that the replisome is an important target of checkpoint regulation, possibly ensuring the coordination between DNA synthesis and replisome progression [21, 27–29], although the details of such regulation are still poorly understood.

In addition to phosphorylation by the checkpoint kinases, ubiquitylation and SUMOylation also play a key role in the response to replication forks stalling. One of the best-characterised targets of ubiquitylation in response to DNA damage is the PCNA clamp, which is mono-ubiquitylated by Rad6-Rad18 and poly-ubiquitylated by Rad5-Ubc13-Mms2. These modifications regulate the post-replicative repair of gaps left behind replication forks and promote either DNA damage tolerance (mono-ubiquitylation) or error-free repair and sister chromatid recombination (poly-ubiquitylation) [30]. Similarly, SUMOylation promotes cell viability in response to replication defects. Budding yeast contains a simple SUMOylation pathway, with a single E2 SUMO-conjugating enzyme and three PIAS-family E3 SUMO ligases, namely Siz1, Siz2 and Mms21 [31, 32]. Mutation of the E2 conjugating enzyme Ubc9 results in sensitivity to treatment with MMS and HU, as does mutation of the E3 ligase *MMS21*, or the simultaneous deletion of *SIZ1* and *SIZ2* [32–34].

Many targets of SUMOylation have been characterised in response to MMS treatment, such as the helicase Sgs1, the Cohesin subunit Scc1 or the recombination factor Rad52 [35–43]. Abolishing the SUMOylation of these targets leads to changes in the dynamics and location of DNA repair, chromosome instability and loss of viability. Moreover, SUMO-ligases localise to sites of ssDNA accumulation and modify proteins recruited nearby, in a manner that is mainly driven by proximity to the damage or to the recombination intermediates [44]. The role of such large-scale modification appears to favour protein-protein interactions amongst repair proteins, thus hastening the kinetics of repair. Therefore, waves of SUMOylation act as a molecular glue that favour DNA damage repair. Interestingly, several replisome proteins and other factors at forks are similarly modified in response to MMS raising the possibility that a similar function might occur at forks [33].

Together, the checkpoint response and protein SUMOylation both ensure the maintenance of cell viability following DNA damage. Interestingly, several observations suggest some cross-talk between these pathways. In fact, SUMOylation regulates the S phase and DNA damage checkpoints. For example, in budding yeast, Mec1, Tel1 Rad9 and Mrc1 are all SUMOylated in response to MMS treatment; in human cells, ATRIP is SUMOylated, thus boosting its binding to ATR, RPA70, TopBP1, and the MRE11-RAD50-NBS1 complex [44, 45]. Similarly, ATR SUMOylation increases its catalytic activity [46].

Conversely, the S phase checkpoint also modulates the SUMOylation response. In budding yeast, Mec1 and Tel1 checkpoint kinases phosphorylate Mms21, and stimulate the SUMOylation of Snf1 [47, 48]. Similarly, human FANCI and FANCD2 are SUMOylated in response to replication fork stalling in an ATR-dependent manner [49]. In contrast, SUMOylation of many proteins is increased in the absence of budding yeast Mec1 [33]. Similar results have been observed in human cells following the inhibition of ATR [50]. It remains to be determined whether this increase reflects a direct inhibitory role of the checkpoint response on SUMOylation, or else it is a consequence of increased DNA damage in the absence of the checkpoint kinases. Nevertheless, the level and the regulation of the cross-talk between the checkpoint response and protein SUMOylation are still understood poorly.

Here we report a site-specific SUMOylation of the Pol2 catalytic subunit of Pol ε, which depends on the Smc5/6 complex and is stimulated robustly in response to the activation of the S phase checkpoint by fork stalling with hydroxyurea (HU). Moreover, we map a SUMO interacting Motif at the C-terminal of Pol2. Our findings suggest a model for the possible regulation of Pol2 by SUMOylation following replication stress.

## Results

### Pol2 is mono-SUMOylated in response to nucleotide depletion

**Fig 1:**
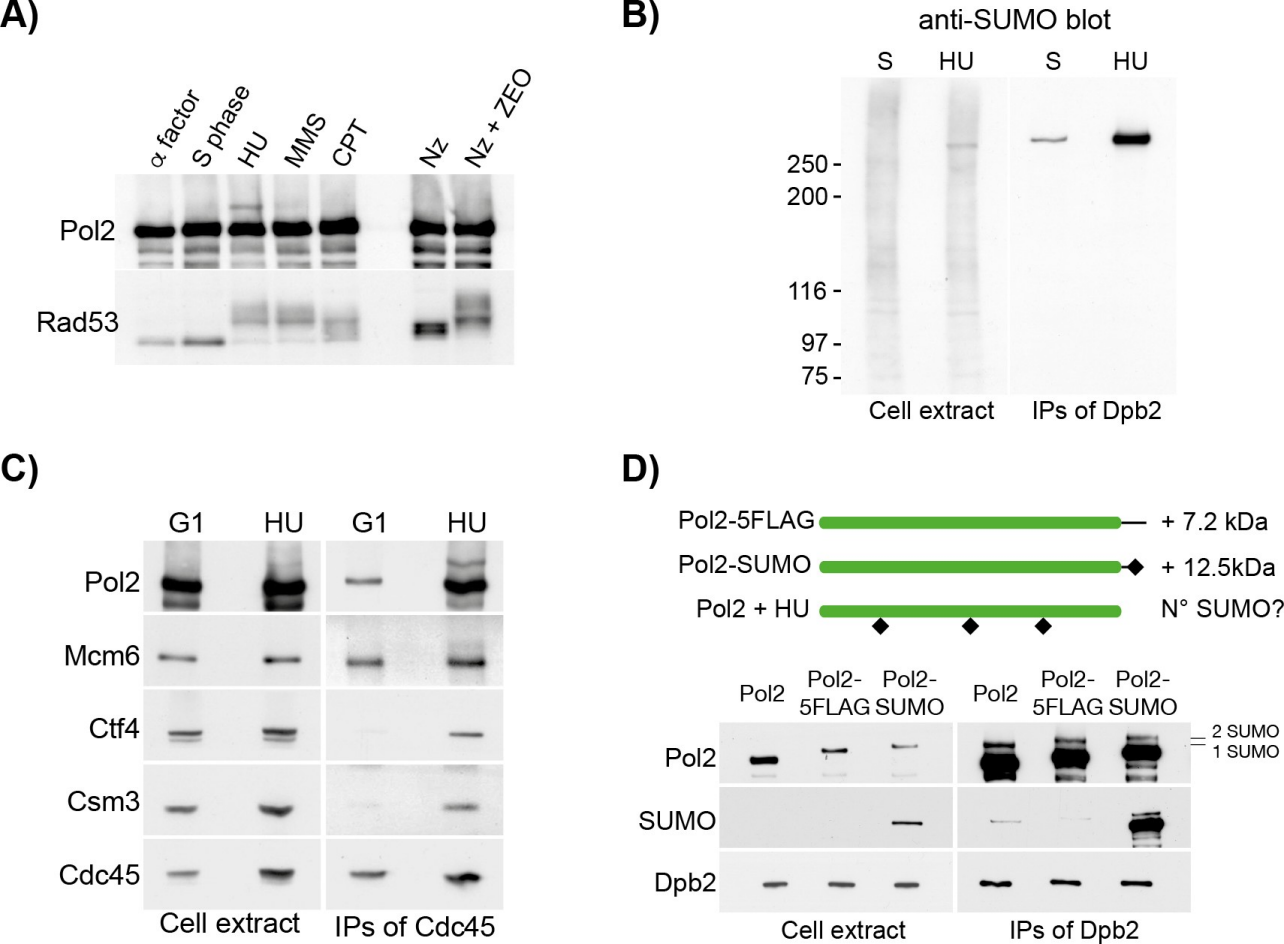
Pol2 is mono-SUMOylated on chromatin in response to nucleotide depletion. **A)** Pol2 is post-translationally modified in response to replication stress, especially following treatment with HU. Cells were grown to the exponential phase, arrested in G1 and synchronously released in S phase for 30 min in YPD (S phase), or for 90 min in medium containing 0.2 M HU (HU), 0.033% methyl methanesulphonate (MMS) or 20 μM Camptothecin (CPT). Exponentially growing cells were also arrested at the G2/M phase with nocodazole, and incubated in the absence (Nz) or in the presence of 70 μM Zeocin (Nz+NEO) for 90 min. Rad53 and Pol2 immunoblotting are shown. **B)** Pol2 is SUMOylated in response to HU. Cells carrying a TAP-tagged version of Dpb2 were grown to exponential phase, arrested in G1 and synchronously released in S phase for 30 min in YPD (S phase) or for 90 min in YPD 0.2 M HU (HU). Pol ε was purified under stringent conditions (700 mM potassium acetate) and the immunoprecipitated material was eluted by TEV cleavage of the TAP tag. Cell extracts and IPs were probed with an anti-SUMO antibody. **C)** SUMOylated Pol2 is enriched at forks. Cells carrying a FLAG-tagged allele of Cdc45 were grown to exponential phase, arrested in G1 and synchronously released in YPD 0.2 M HU (HU) for 90 min. Proteins were cross-linked with formaldehyde. Cdc45 was immunoprecipitated and protein samples were analysed by immunoblotting. **D)** Pol2 is mono-SUMOylated. (Top). Schematic representation of the tagged alleles of *POL2* used. (Bottom). Cells carrying a TAP-tagged version of Dpb2 and either a wild type, a FLAG-tagged or a SUMO-tagged versions of *POL2* were grown to exponential phase, arrested in G1 and synchronously released in YPD 0.2 M HU for 90 min. Dpb2 was then purified and analysed by immunoblotting.

Whilst searching for novel post-translational modifications of the replisome (De Piccoli et al, 2013; Maric et al 2014), we discovered that the Pol2 catalytic subunit of Pol ε was modified in response to nucleotide depletion by hydroxyurea (HU, Fig 1A). Interestingly, this effect was preferentially seen in response to nucleotide depletion, compared to what observed in cells incubated with the alkylating agent methylmethane sulphonate (MMS), or the topoisomerase I poison Camptothecin (CPT). Moreover, the induction of double strand breaks outside of S phase with Zeocin treatment did not promote Pol2 modification (Fig 1A).

After isolation of Pol ε by immunoprecipitation of the Dpb2 subunit, we observed that modified Pol2 was resistant to phosphatase treatment and was not recognised by antibodies to ubiquitin (S1A and S1B Fig). In contrast, modified Pol2 was recognised by antibodies specific for yeast SUMO (Fig 1B) and represented one of the most abundant SUMO conjugates in the cell extracts. Moreover, SUMOylated Pol2 could also be detected in untreated S phase cells, though to a 10-fold lower level than after HU treatment (Fig 1B, S1C and S1D Fig).

To address whether Pol2 SUMOylation occurs at replication forks or in the fraction not associated with DNA, we treated cells with formaldehyde to cross-link proteins to DNA, and then made extracts containing high salt and detergents, so that any observed complexes would reflect the *in vivo* situation, rather than interactions occurring *ex vivo* [21]. In this way, we observed that the fraction of SUMOylated Pol2 was greatly enriched after immunoprecipitation of Cdc45, indicating that Pol2 SUMOylation occurs in the context of the replisome at replication forks (Fig 1C).

Finally, we compared the size of the observed Pol2-SUMO band with markers representing Pol2-5FLAG (7.2 kDa larger than wild type Pol2) or a linear fusion of Pol2 and a non-conjugatable form of the yeast SUMO Smt3 (12.5 kDa larger than wild type Pol2). After immunoprecipitation of Pol ε from cells treated with HU, the SUMOylated form of wild type Pol2 was seen to migrate equivalently to the Pol2-Smt3 fusion protein (Fig 1D), indicating that Pol2 is mono-SUMOylated in response to nucleotide depletion. Taken together, these findings indicate that Pol2 is mono-SUMOylated at replication forks; this modification occurs at a low level during unchallenged DNA replication, and is greatly stimulated in response to nucleotide depletion.

### Pol2 is the main target of SUMOylation within the core replication machine

Previous work showed that DNA damage induces a wave of protein SUMOylation that affects many proteins and works as a “molecular glue” to promote protein interaction and thus increase the kinetics of repair and favour cell growth [44]. Similarly, many replication proteins, including Pol2, show a basal level of SUMOylation in S phase (Fig 1B) and are SUMOylated in response to high levels of MMS [33]. In contrast, in our experiments, anti-SUMO immunoblots of whole cell extracts indicated that a single band was prominent after HU treatment, corresponding to Pol2-SUMO (Fig 1B, left panel). This suggested that increased SUMOylation of Pol2 in response to nucleotide depletion might be specific and not be part of a SUMOylation wave of all the neighbouring proteins.

**Fig 2:**
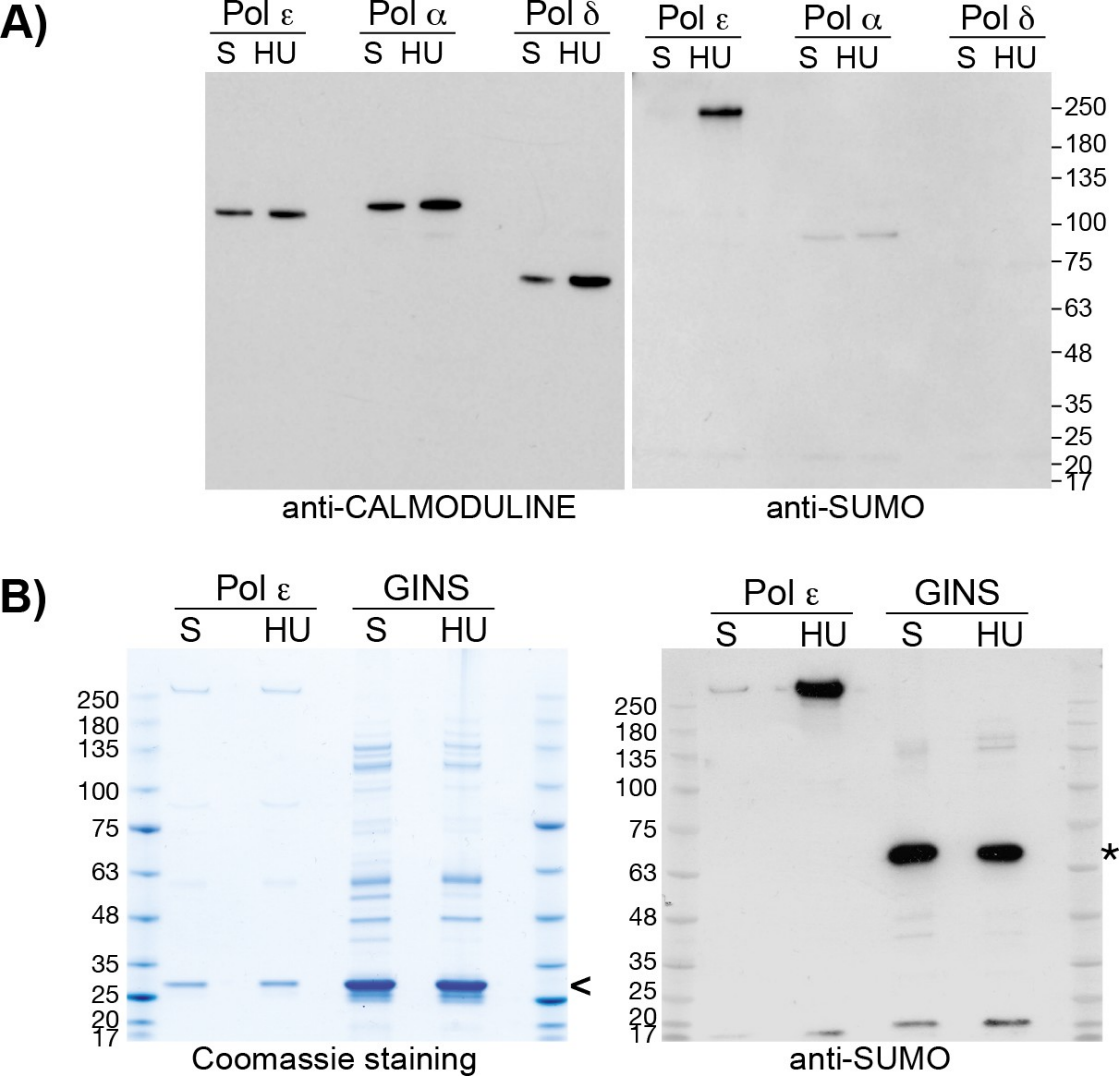
Pol2 is the major target of SUMOylation within the replisome in response to nucleotides depletion. **A)** Pol2 is the major SUMOylation substrate among the replicative DNA polymerases in response to HU. Cells carrying a TAP-tagged version of Dpb2 (Pol ε), Pol12 (Pol α) or Pol31 (Pol δ) were grown to the exponential phase, arrested in G1, and synchronously released in S phase in the absence (S) or in the presence of 0.2 M HU for 30 min and 90 min, respectively. The TAP-tagged proteins were immunoprecipitated under stringent conditions, eluted by TEV cleavage of the TAP tag and analysed by immunoblotting. The samples were also analysed by mass spectrometry and shown to co-purify all components of the three polymerases (S2 Fig). **B)** Pol2 is the main target of SUMOylation within the replisome in response to HU. Cells carrying a TAP-tagged version of Dpb2 (Pol ε) or Sld5 (GINS) were synchronously released in the absence (S) or in the presence of 0.2 M HU for 30 min and 90 min, respectively. The TAP-tagged proteins were immunoprecipitated from 2.5*10^9^ (Dpb2-TAP) and 10^10^ cells (TAP-Sld5) in 300mM potassium acetate, eluted by TEV cleavage of the TAP tag and (for the Sld5 IPs) concentrated by TCA precipitation. TAP-Sld5 purification allows to isolate the Replisome Progression Complex, comprising of the CMG helicase and other regulatory factors at the replication fork (114). Protein samples were separated by electrophoresis and Coomassie-stained or analysed by immunoblotting. The symbol **<** indicates the TEV proteinase band, the symbol * indicates the un-cleaved form of TAP-Sld5.

To explore this further, we tested directly whether other DNA polymerases were also SUMOylated following HU treatment. Cells carrying TAP-tagged alleles of the second largest subunits of the three replicative DNA polymerases (namely *POL12*-*TAP*, *POL31-TAP* and *DPB2-TAP* for Pol α, Pol δ and Pol ε, respectively) were arrested in G_1_ and then released into S phase in the presence or absence of HU. After immunoprecipitation and stringent washing, the polymerase complexes were released from beads by TEV cleavage and analysed by mass spectrometry (S2 Fig, confirming the presence of all predicted subunits) and immunoblotting with antibodies to the residual portion of the TAP-tag (Fig 2A, left panel) or yeast SUMO (Fig 2A, right panel). We observed that Pol2 was the only polymerase subunit to be SUMOylated under these conditions, and this was greatly increased in the presence of HU. Furthermore, we analysed replisome material that was isolated by immunoprecipitation of TAP-tagged Sld5, a component of the GINS complex and the CMG helicase. No further SUMOylated proteins were detected in HU-treated cells, (Fig 2B). All together, these data show that Pol2 is a preferential target of SUMOylation within the core replication machine, in response to nucleotide depletion, and indicate that Pol2 SUMOylation does not occur in the context of a ‘promiscuous wave’ of general SUMOylation at replication forks under these conditions, contrasting with the response to double strand breaks and MMS treatment.

### Pol2 hyper-SUMOylation in HU is dependent on the S phase checkpoint

**Fig 3:**
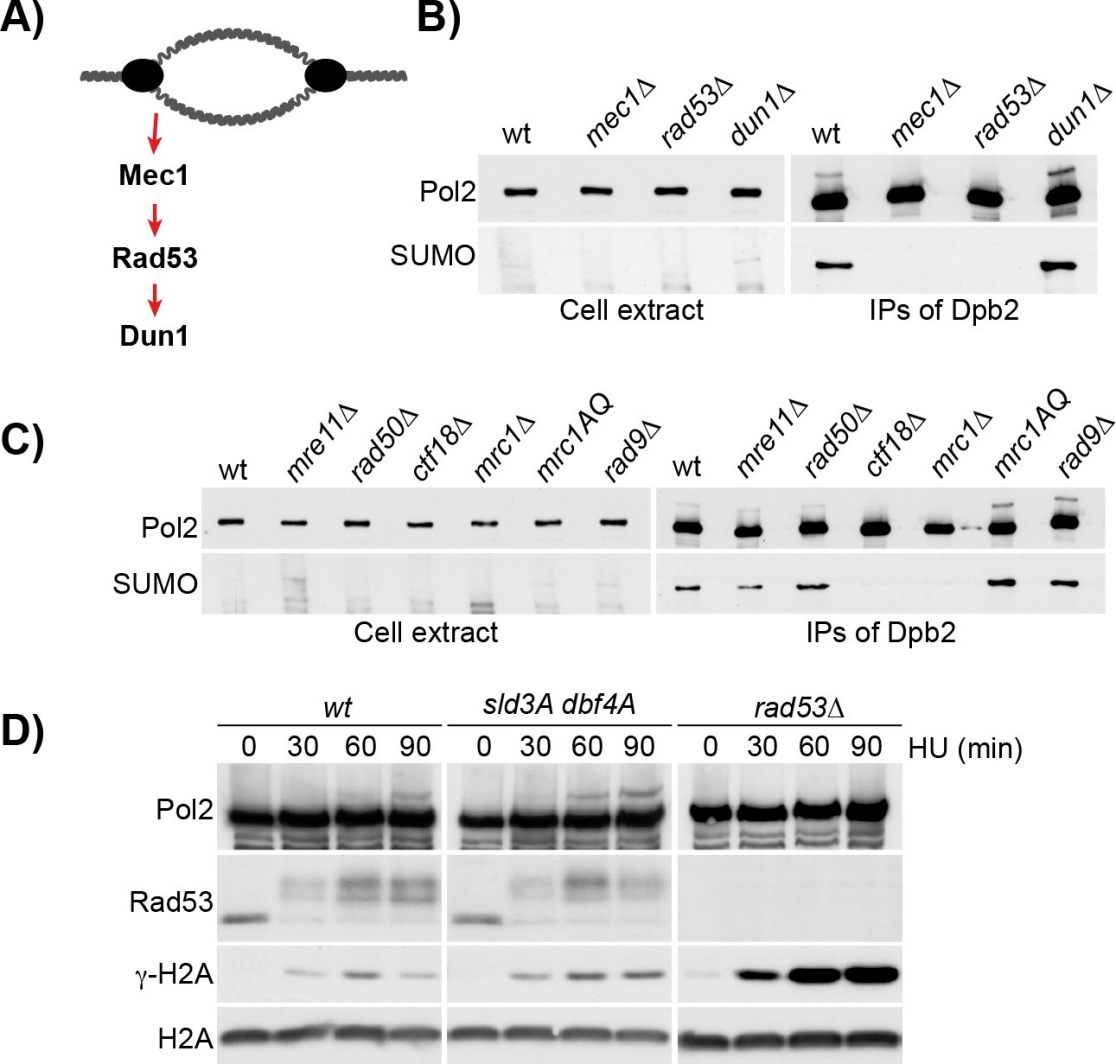
Pol2 SUMOylation depends on Rad53 and S phase checkpoint mediators. **A)** Representation of the checkpoint kinases cascade**. B)** Pol2 SUMOylation depends on the checkpoint kinases Mec1 and Rad53. Strains *sml1Δ* (WT), *sml1Δ mec1Δ* (*mec1Δ*), *sml1Δ rad53Δ* (*rad53Δ*) and *sml1Δ dun1Δ* (*dun1Δ*), all carrying a TAP-tagged version of DPB2, were grown to the exponential phase, arrested in G1 and synchronously released in medium containing 0.2 M HU for 90 min. Dpb2 was immunoprecipitated and the protein samples were analysed by immunoblotting. **C)** Pol2 SUMOylation depends on the S phase checkpoint mediators Mrc1 and Ctf18. Strains deleted for genes required for the activation of Rad53 in response to double strand breaks (*mre11Δ*, *rad50Δ*), to replication stress, either through the S phase checkpoint (*mrc1Δ*, *ctf18Δ*, *mrc1-AQ*), or the DNA damage checkpoint (*rad9Δ*), were grown to the exponential phase, arrested in G1 and synchronously released in medium containing 0.2 M HU for 90 min. Pol ε was immunoprecipitated via Dpb2-TAP and analysed by immunoblotting. **D)** Analysis of the kinetics of Pol2 SUMOylation. Wild type cells, mutants *sld3-37A dbf4-4A*, and *sml1Δ rad53Δ* were grown to the exponential phase, arrested in G1 and synchronously released in medium containing 0.2 M HU for 90 min. Cells were collected every 30 min, and proteins were analysed by TCA extraction and immunoblotting. Pol2 SUMOylation appears after Rad53 activation and it’s more pronounced in cells defective in the inhibition of late origin firing.

To investigate whether Pol2 SUMOylation following nucleotide depletion is dependent upon the S phase checkpoint, we initially monitored Pol2 SUMOylation in cells lacking the yeast checkpoint kinases Mec1, Rad53 and Dun1 (Fig 3A). As described above, Pol2 SUMOylation was detected in control cells, as well as in *dun1Δ* cells (Fig 3B). However, increased levels of SUMOylation of Pol2 were not detected in cells lacking *MEC1* or *RAD53* (Fig 3B). Since Mec1 and Dun1 are respectively upstream and downstream of Rad53 activation [51, 52], we conclude that Pol2 SUMOylation is Rad53-dependent. Consistent with this hypothesis, Pol2 SUMOylation in HU is dependent upon Mrc1 and Ctf18, both mediators of Rad53 activation at forks [53–55], but was independent of the DNA damage checkpoint mediator Rad9 [56, 57] or the dsDNA break response factor Mre11 [58, 59] (Fig 3C, S3A Fig). Moreover, the dependency on the S phase checkpoint for the increase in SUMOylation was observed mainly in HU (S3B Fig). Interestingly, we observed that the *mrc1-AQ* allele did not affect the SUMOylation of Pol2, suggesting that the delay in Rad53 checkpoint activation in this background was insufficient to abolish the S phase checkpoint response, as previously observed [54].

Finally, we compared Pol2 SUMOylation with the kinetics of checkpoint activation, as reflected by Rad53 hyper-phosphorylation. We observed that Pol2 SUMOylation lags behind the hyper-phosphorylation of Rad53, consistent with Pol2 SUMOylation being downstream of checkpoint activation (Fig 3D). Moreover, the fraction of Pol2 that is SUMOylated was increased in *dbf4-4A sld3-37A* cells which cannot restrain late origin firing in HU [60], consistent with the observation that Pol2 SUMOylation mostly occurs at replication forks (Fig 3D). All together, we conclude that Pol2 is SUMOylated as part of the replisome, dependent upon activation of the Rad53 kinase by the S phase checkpoint pathway.

### Pol2 SUMOylation requires the Mms21 SUMO-ligase subunit of the Smc5/6 complex

In budding yeast cells, the SUMOylation pathway depends on a single E1 SUMO-activating enzyme Aos1/Uba2, a single E2 SUMO-conjugating enzyme Ubc9, and three E3 SUMO-ligases, namely Siz1, Siz2 and Mms21 [31, 61]. Whereas Siz1 and Siz2 are not needed for cell viability, Mms21 is essential, although this reflects other roles in addition to its SUMO-ligase activity [34, 62, 63]. Mms21 (or Nse2) is part of the conserved Smc5/6 complex, an 8-subunits complex that safeguards genome stability in response to double strand breaks or obstacles to replication fork progression, and regulates the topological status of chromosomes during and following DNA replication [32, 38, 64–70].

**Fig 4:**
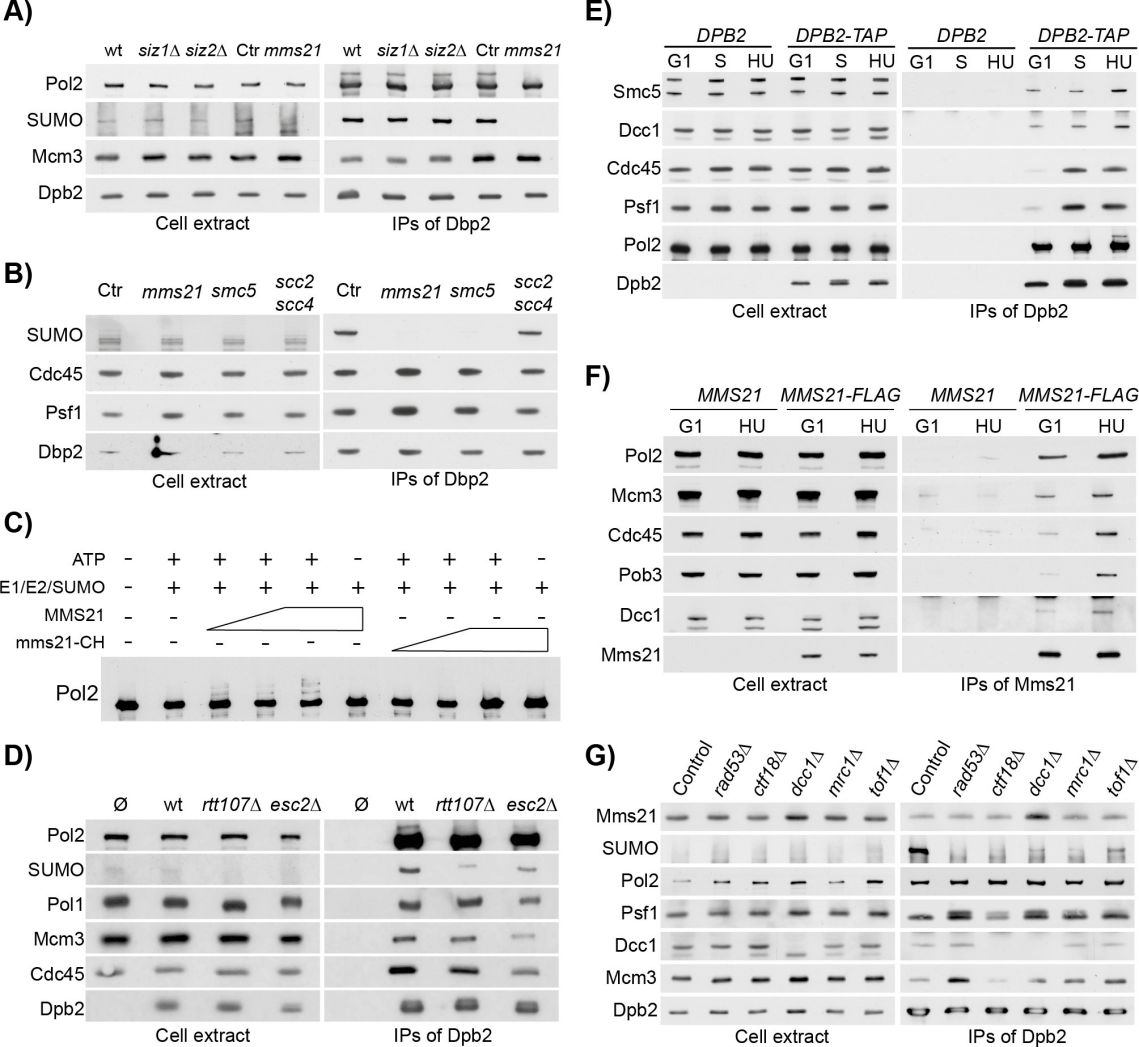
Pol2 is SUMOylated by Mms21 and interacts with the Smc5/6 complex. **A)** Pol2 SUMOylation depends on the E3-SUMO ligase Mms21. Wild type, *siz1Δ* and *siz2Δ* cells were grown to the exponential phase, arrested in G1 and synchronously released in medium containing 0.2 M HU for 90 min. In addition, a control strain (Ctr) and one carrying an auxin-inducible degron allele of *MMS21* (*mms21*), were grown in YPRaf to the exponential phase, arrested in G1, resuspended in YPGal for 35 min, incubated for 60 min in medium containing a final concentration of 0.5 mM indole-3-acetic acid (IAA) to induce protein degradation, and released in YPGal containing 0.2 M HU and 0.5 mM IAA for 90 min. Dpb2-TAP was immunoprecipitated and protein samples were analysed by immunoblotting. **B)** Smc5/6 complex is required for Pol2 SUMOylation. Strains wild type, *mms21-aid* (*mms21*), *smc5-aid* (*smc5*), *scc2-aid scc4-aid* (*scc2 scc4*) and a control strain were grown in YPRaf to the exponential phase, arrested in G1, resuspended in YPGal for 35 min, incubated for 60 min in medium containing a final concentration of 0.5 mM IAA, and released in YPGal medium containing 0.2 M HU and 0.5 mM IAA for 90 min. Dpb2-TAP was immunoprecipitated and protein samples were analysed by immunoblotting. **C)** Pol2 is SUMOylated *in vitro* by Mms21. Pol ε and the Smc5/6 complex—either carrying a wild type allele of *MMS21* or the SUMO-ligase defective *mms21-(C200A H202A)* mutant, (referred to as *mms21-CH*, also see S4B Fig)—were purified at high salt conditions (700 mM potassium acetate) via a TAP tag (on Dpb2 and Smc5 respectively). SUMO (Smt3GGΔ), the E1 SUMO-activating enzymes Aos1/Uba2 and the E2 SUMO-conjugating enzyme Ubc9 were purified from *E*. *coli*. The *in vitro* SUMOylation reaction was conducted for 60 min at 30°C. **D)** Pol2 SUMOylation partially depends on *RTT107* and *ESC2*. An untagged strain (Ø), or wild type, *rtt107Δ* and *esc2Δ* cells carrying a TAP-tagged allele of *DPB2*, were grown to the exponential phase, arrested in G1 and synchronously released in medium containing 0.2 M HU for 90 min. Dpb2-TAP was immunoprecipitated and protein samples were analysed by immunoblotting. **E)** Smc5 co-immunoprecipitates with Pol ε in G1 and S phase. Cells carrying either a tagged or untagged version of Dpb2 were grown to exponential phase, arrested in G1 and released in YPD for 30 min (S) or in YPD 0.2 M HU for 90 min (HU). Dpb2-TAP was immunoprecipitated and protein samples were analysed by immunoblotting. **F)** Pol2 co-immunoprecipitates with Mms21. Cells carrying a tagged or untagged version of Mms21 were arrested in G1 and released in YPD 0.2 M HU for 90 min (HU). Mms21-5FLAG was immunoprecipitated and protein samples were analysed by immunoblotting. **G)** Mms21 interaction with Pol ε does not depend on S phase checkpoint components. Wild type cells and *sml1Δ rad53Δ*, *mrc1Δ*, *tof1Δ*, *ctf18Δ* and *dcc1Δ* mutants, carrying a tagged version of Dpb2, were arrested in G1 and synchronously released either in YPD for 30 min (S) or in medium containing 0.2 M HU for 90 min (HU). Dpb2-TAP was immunoprecipitated and protein samples were analysed by immunoblotting.

To investigate which SUMO E3 ligases might be required for Pol2 SUMOylation, we isolated Pol ε from cells lacking Siz1 or Siz2, or from cells after rapid depletion of Mms21 via an auxin degron cassette [71]. As shown in Fig 4A, Pol2 SUMOylation was unaffected in *siz1Δ* or *siz2Δ* cells, but was abolished in the absence of Mms21 (Fig 4A). This indicated that the Smc5/6 complex, of which Mms21 is a subunit, drives Pol2 SUMOylation. Correspondingly, Pol2 SUMOylation was prevented by depletion of a degron-tagged version of Smc5, (Fig 4B). Interestingly, depletion of the Scc2-4, the loading complex for Smc5/6 [65, 68] did not abolish Pol2 SUMOylation (Fig 4B), despite blocking cell cycle progression and causing loss of cell viability (S4A and S4B Fig), suggesting that at least some of the functions of Smc5/6 complex at stalled forks are independent of Scc2-4. Finally, the basal level of SUMOylation observed in S phase also depended on Mms21, but not Rad53, suggesting that the latter is only needed for the 10-fold increase in SUMOylation observed in HU (S3B Fig, S5A and S5B Fig).

To explore whether Mms21 directly SUMOylates Pol2, we first mutated the sp-RING (Siz1/PIAS RING) domain of Mms21, which binds the Ubc9 E2 enzyme for SUMOylation. Pol2 SUMOylation was abolished in the *mms21-C200A H202A* sp-RING mutant (*mms21-CH*), indicating that Pol2 SUMOylation requires the E3 ligase activity of Mms21 (S5C Fig). We then tested whether Mms21 was able to directly SUMOylate Pol2 *in vitro*. To this end, we purified Pol ε, Smc5/6-Mms21 and Smc5/6-mms21-CH from budding yeast cells, and then incubated them with recombinant budding yeast E1, E2 and SUMO proteins that were purified from *E*. *coli*. *In vitro* SUMOylation of Pol2 depended not only on the E1 and E2 enzymes, but also upon the RING domain of Mms21 (Fig 4C, compare titrations of Mms21 and Mms21-CH). Similarly, expression of wild type Mms21 was sufficient to drive *in vivo* Pol2 SUMOylation in *mms21-CH* cells arrested in HU (S5D Fig). These findings indicate that Pol2 is a direct target of the Mms21-Smc5/6 E3 SUMO ligase, both *in vitro* and *in vivo*.

Previous studies indicated that SUMOylation of target proteins by Mms21 depends not only on the integrity of the Smc5/6 complex, but also on the binding to the BRCT-containing protein Rtt107, at least in response to MMS treatment [56, 72]. Moreover, deletion of *ESC2*, a regulator of recombination containing SUMO-like domains, affects the SUMOylation of several Mms21 targets, in both budding and fission yeasts [73–75]. To test whether *RTT107* and *ESC2* were required for the SUMOylation of Pol2 in HU, wild type, *rtt107Δ* and *esc2Δ* cells were incubated in HU, before immunoprecipitation of and immunoblotting of Pol ε. We observed that Pol2 SUMOylation was decreased in the absence of either Rtt107 or Esc2 (Fig 4D), suggesting that these factors promote robust SUMOylation.

### Pol2 co-purify with Smc5/6

Since the SUMOylation of Pol2 was exclusively dependent on Mms21, we tested whether Pol ε associates with the Smc5/6-Mms21 E3 ligase complex, using strains with tagged versions of Dpb2, Smc5 or Mms21. Cells were arrested in G1 phase and then released synchronously into S phase in the presence or absence of 0.2 M HU. As shown in Fig 4E and 4F and S6A and S6B Fig, Smc5 and Mms21 co-purified with Pol ε throughout the cell cycle, regardless of whether cells had been treated with HU to activate the S phase checkpoint pathway. These observations were reminiscent of previous data showing that the Ctf18 complex interacts with Pol ε throughout the cell cycle [76–78]. Moreover, the association of Pol ε with Smc5/6-Mms21 and Ctf18-RFC showed a similar salt sensitivity (S6C Fig). However, the association of Smc5/6-Mms21 with Pol ε was independent of Ctf18-RFC (Fig 4G).

To confirm that Smc5/6-Mms21 interacts with Pol ε independently of the S phase checkpoint pathway, we showed that their association still persisted in cells lacking the checkpoint kinases Rad53 or Mec1 (Fig 4G, S6D Fig). Moreover, the interaction between Smc5/6-Mms21 and Pol ε did not require the SUMO-ligase activity of Mms21 (S6D Fig). All together, these data indicate that the association of Pol ε with the Smc5/6-Mms21 complex is independent of other replisome components and does not depend on the S phase checkpoint.

### Pol2 is SUMOylated at K571

**Fig 5:**
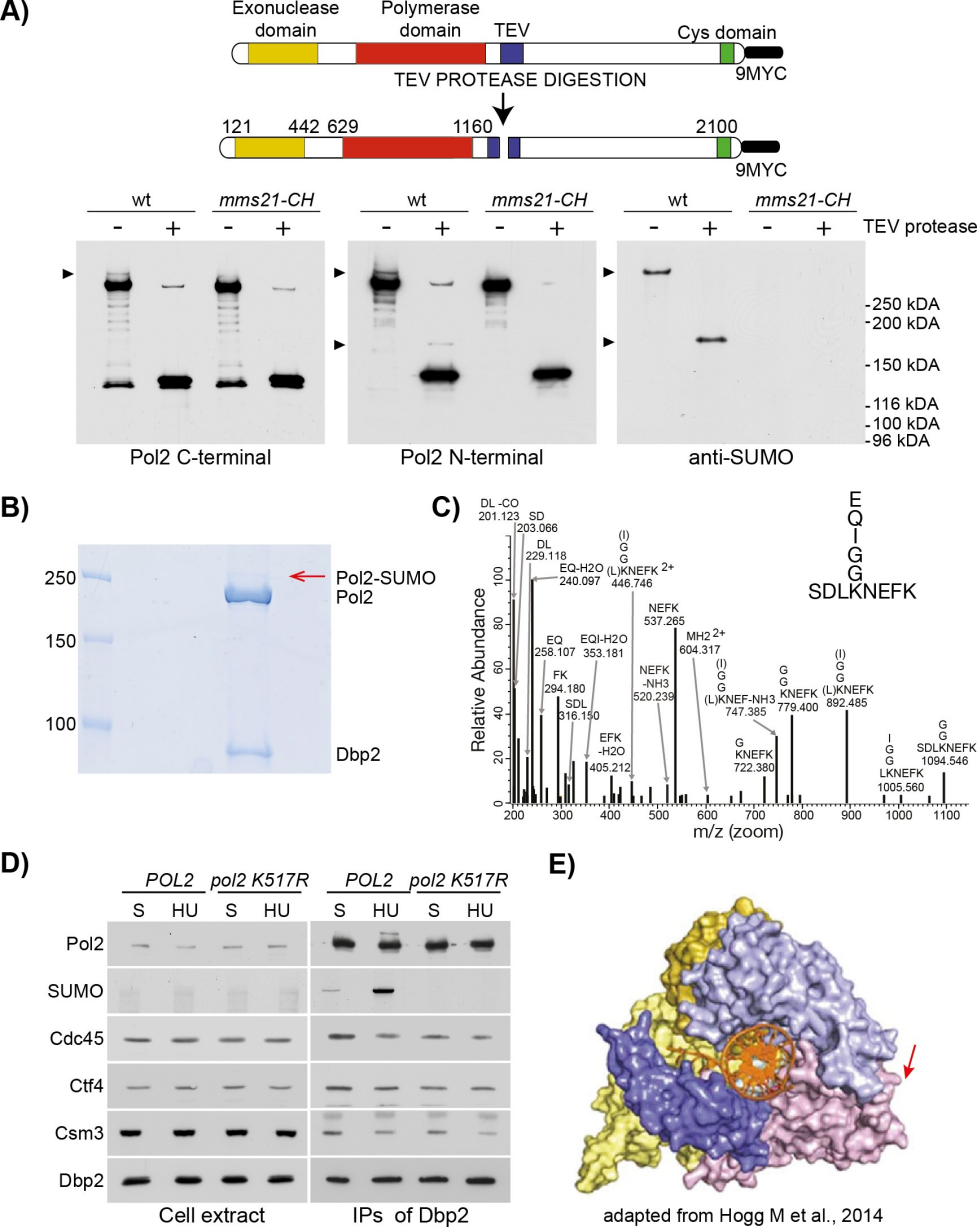
Pol2 is SUMOylated at K571. **A)** Mms21 SUMOylates Pol2 N-terminal half. (Top). A graphic representation of the Pol2 allele used in the experiment is shown. *POL2* was tagged at the C-terminal with a 9MYC tag. In addition, a 3xTEV sequence was inserted at the position S1227. (Bottom). Wild type and *mms21-CH* mutant cells, carrying the *POL2-(3TEV)-9MYC* allele, were grown to exponential phase, arrested in G1 and synchronously released in YPD 0.2 M HU for 90 min. Pol2 was then immunoprecipitated, incubated in the presence or absence of the 20 units of TEV protease, eluted by boiling and analysed by immunoblotting. The N-terminal part of Pol2 was detected with a polyclonal antibody raised against the N-terminal half of Pol2, while the C-terminal half was examined with an anti-MYC antibody. **B)** Mass spectrometry analysis reveals a single putative site of SUMOylation at Lysine 571. (Left) Cells carrying a Dpb2 TAP-tagged allele were synchronously released in medium containing 0.2 M HU for 90 min. Pol ε was immunoprecipitated at 700 mM potassium acetate and eluted by TEV cleavage. Samples were analysed by electrophoresis and stained in Coomassie blue. The Pol2-SUMO band was cut and digested with trypsin for mass spectrometry analysis. **C)** MS/MS spectrum showing Pol2 modification at K571 by SUMO. **D)** Pol2 SUMOylation is abolished in a K571R mutant. Cells carrying a DPB2 TAP-tagged allele and a wild type or a *pol2 K571R* (*pol2KR*) allele were grown to exponential phase, arrested in G1 and synchronously released either in fresh medium for 30 min (S) or in YPD 0.2 M HU for 90 min (HU). Dpb2-TAP was immunoprecipitated and analysed by immunoblotting. **E)** K571 is in the palm domain of Pol2. Illustration of the position of K571 within Pol2 N-terminal crystal structure (81). K571 is in a Pol2-specific large insertion within the palm domain.

To map the SUMOylation site(s) within Pol2, we first investigated whether the modification occurs within the amino-terminal half of the protein that contains the active polymerase domain, or maps to the non-catalytic carboxy-terminal half, or both. To this end, we modified the *POL2* locus in yeast cells to introduce 3xTEV sites within the flexible unstructured region in the middle of the protein, and also added a C-terminus 9MYC tag. We used the latter to purify TEV-cleavable Pol ε from HU-arrested cells, and then analysed the products of TEV cleavage with antibodies specific to the N-terminus of Pol2 or to the C-terminal 9MYC tag. Whereas the Pol2 C-terminal half was unmodified, the N-terminal half of the protein showed an extra band that was dependent upon the E3 ligase activity of Mms21 and recognised by anti-SUMO antibodies (Fig 5A). This indicates that Pol2 is SUMOylated within the N-terminal half of the protein that contain the conserved catalytic exonucleolytic and polymerase domains [79]. To map the specific site of modification, we used mass spectrometry analysis of Pol ε purified from HU-treated cells (Fig 5B). This identified a single SUMOylated residue at Lysine 571 (Fig 5C), which falls within a predicted consensus site for SUMOylation (Ψ-K-X-E, where ψ is a hydrophobic amino acid, and X any amino acid residue [80]). To test if K571 is indeed the only site of Pol2 SUMOylation under these conditions, we modified the *POL2* genomic locus to produce the *pol2 K571R* allele. This was sufficient to abolish Pol2 SUMOylation in HU-treated cells, as well as the basal SUMOylation observed in S phase (Fig 5D), indicating that K571 is the only site of modification. Analysis of the structure of the Pol2 catalytic domain showed that K571 is located on the outer surface of the protein, in a Pol ε-specific insertion within the palm domain of the Polymerase B family [81] (Fig 5E).

### Pol2 C-terminal has a SUMO-binding motif

**Fig 6:**
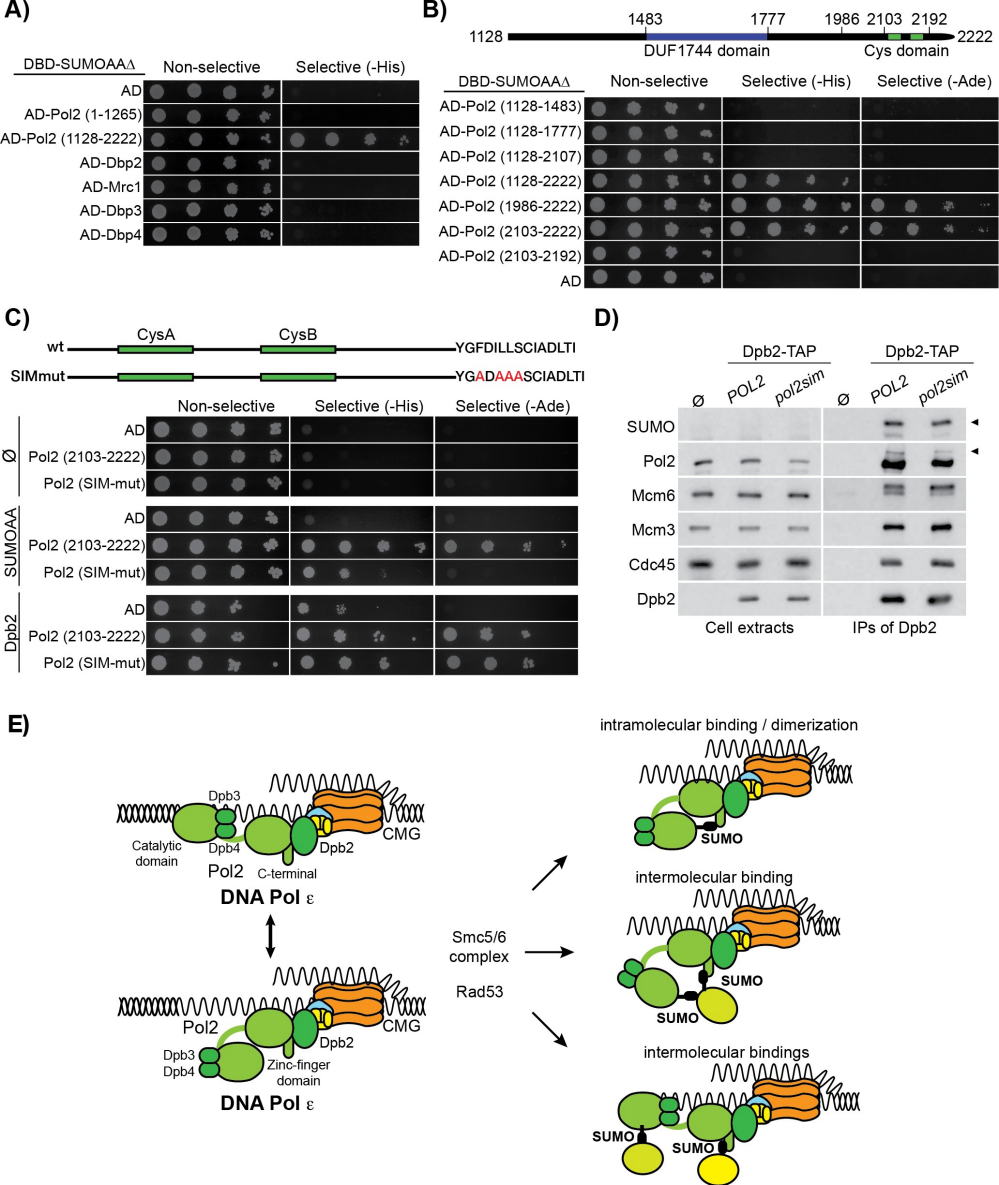
Pol2 C-terminal contains a SUMO-interacting motif (SIM). **A)** The C-terminal half of Pol2 interacts with SUMO. The ability of the subunits of Pol ε- Dpb2, Dpb3, Dpb4, Pol2 N-terminal (1–1265) and Pol2 (1128–2222)—and of Mrc1 to interact with SUMO (Smt3AAΔ, an allele that cannot be used as a moiety for SUMOylation) was tested by using the yeast two-hybrids assay. Pol2 C-terminal shows the ability to interact with SUMO. **B)** Mapping of the interaction between several fragments of Pol2 C-terminal half and SUMO was tested by yeast two-hybrids assay. Pol2 (2013–2222) fragment is sufficient for the interaction with SUMO, while the deletion of the last 30 amino acids blocks the binding. **C)** Identification of a SIM motif at the extreme C-terminal of Pol2. The analysis of the sequence identified a putative SIM sequence at the extreme C-terminal. Mutation of this sequence abolished the binding of the SUMO protein while not affecting the interaction with Dpb2. **D)** Pol ε composition and SUMOylation is not affected by the mutation of the SIM motif at the C-terminal. An untagged strain, *POL2* or *pol2sim* cells carrying a TAP-tagged allele of *DPB2*, were grown to the exponential phase, arrested in G1 and synchronously released in YPD 0.2 M HU for 90 min. Dpb2-TAP was immunoprecipitated and protein samples were analysed by immunoblotting. **E)** Possible models of action of Pol2 SUMOylation in response to replication stress. Rad53, activated through the S phase checkpoint, and the E3 SUMO-ligase Mms21, interacting with the Pol ε, promote the SUMOylation of Pol2 at Lysine 571. This SUMOylation might then lead to (top) an intramolecular binding of SUMO by Pol2 SIM (or dimerization), (middle) recruitment of a SUMOylated factor at forks, or (bottom) recruitment of different proteins by the SUMO and SIM sequences.

Protein SUMOylation often regulates protein-protein interactions [31, 82, 83], such as the binding of the anti-recombinogenic helicase Srs2 to SUMOylated PCNA [84, 85]. In such cases, SUMO usually interacts in a non-covalent manner with a SUMO-Interacting-Motif (SIM) in the binding partner [86]. We were unable to identify novel partners of SUMOylated Pol2 by mass spectrometry analysis of HU-arrested cells; we therefore tested, via yeast two-hybrid assays, whether Pol ε or any of its known interactors might bind to SUMO (Pol2-NT, Pol2-CT, Dpb2, Dpb3, Dpb4, Mrc1). We observed that, uniquely amongst the proteins tested, the Pol2 C-terminal half interacted with SUMO, (Fig 6A, S7A Fig). Subsequent truncations identified the last 119 amino acids of Pol2 as sufficient for SUMO binding (Fig 6B, S7B Fig). This region contains two conserved cysteine motifs (CysA and CysB), believed to co-ordinate either zinc ions or iron-sulfur clusters [87, 88], neither of which were important for interaction with SUMO (S7C Fig). In contrast, the final 30 amino acids of Pol2 were required for SUMO binding (Fig 6B) and contains the sequence 2210-FDILL -2214 which conforms to the SIM consensus (Ψ-X-Ψ-Ψ-Ψ—with Ψ = hydrophobic, [89]). Mutation of the latter to ADAAA greatly diminished the binding of Pol2-CT to SUMO, without affecting the interaction with Dpb2, indicating that Pol2-CT contains a functional SIM (Fig 6C), distinct from sequences important for assembly of the Pol ε complex [90]. Consistent with this view, we generated a *pol2-sim* strain with mutations in the SIM motif within the endogenous POL2 locus, and found that the resulting protein was incorporated into Pol ε and the replisome, without affecting Pol2 SUMOylation in HU-arrested cells (Fig 6D). All together, this indicates that SUMO might regulate not only the N-terminal part of Pol2 by modification of K571, but also the C-terminal half via the SIM motif.

## Discussion

In budding yeast and mammalian cells, SUMOylation is essential for cell viability and defects in this pathway leads to severe genome instability. Both SUMOylation and non-covalent interaction with SUMO affect a large number of proteins [91–95]; nevertheless, SUMOylation relies on a simple pathway (in particular when compared with the complexity of E2 and E3 enzymes in the ubiquitilation pathway). Whether specificity is important and how it might be achieved is still poorly understood.

Here we show that the catalytic subunit of Pol ε is mono-SUMOylated in a highly specific manner. Following HU incubation, Pol2 is SUMOylated by Mms21 and this depends on Rad53 and the S-phase checkpoint mediators Ctf18 and Mrc1. Whilst at this point we cannot exclude that other factors at fork might be SUMOylated in an Mms21- and Rad53-dependent SUMOylation wave in response to HU, several observations suggest that this might not be the case. First, while a SUMO “glue” model would predict uniform levels of SUMOylation among several replisome components, our direct analysis of the core components of the replication machine provide evidences contrary of this model (Fig 2A and 2B). In addition, amongst the other known Mms21-dependent targets of SUMOylation present at forks such as Sgs1/Top3/Rim1 and Scc1 [96, 97], the post-translational modification is mainly dependent on MMS and is downstream of recombination repair, a process that is actively inhibited by HU [35, 39, 98]. Moreover, following replication stresses, *RAD53* and *MEC1* deletions highly elevate the level of SUMOylation of the targets observed, contrary to what observed for Pol2 [33, 35, 99]. Finally, the ability of Pol ε to interact with Smc5/6 complex independently of other replisome components suggests a specific targeting of the SUMOylation.

While Pol2 is SUMOylated to a basal level during S phase, and this modification does not depend on Rad53 (Fig 1B, S1C Fig, S5B Fig), we observe that fork stalling with HU specifically induces about a ten-fold increase in the levels of SUMOylation of Pol2 in an S phase checkpoint dependent manner. At this point, is still not clear how this increased targeting occurs. In fact, while the basal level of SUMOylation observed in S phase might be explained by the interaction between the Smc5/6 and Pol ε and the stimulatory effect of DNA on Mms21 activity [100](Fig 4E and 4F), the increased levels observed following HU treatment cannot be explained simply by modulation of protein-protein interactions, since we measure neither an increase in affinity between Pol ε and Smc5/6 in HU, nor observe the weakening of the interaction between these two complexes in S phase checkpoint mutants (Fig 5G, S6D Fig). Alternatively, phosphorylation by Rad53 of Pol ε, the Smc5/6 complex or other neighbouring proteins might increase the efficiency of SUMOylation (either by stimulating the catalytic activity of Mms21 or by making K571 a better substrate for modification) or promote the stability of the modification (by sheltering or protecting the modification from SUMO-proteases). While several subunits of the Smc5/6 complex and Pol ε are phosphorylated following HU incubation such as Dpb2, Dpb3, Dpb4, Mms21, Nse4 and Nse6 [22, 47, 101], future work will address whether these are responsible for the SUMOylation of Pol2 in HU.

Our data point to a specific role of Smc5/6 during DNA replication fork stalling, consistent with the localisation of Smc5/6 to sites of BrdU incorporation following HU treatment [68, 102]. Interestingly, the functional importance of Smc5/6 during DNA replication is still not clear: in fact, limiting the expression of Smc5/6 only in late S phase and G2/M can sustain cell viability, suggesting that the complex main function can be limited to post-replicative repair, as observed, for example, for Sgs1 in a *pol32Δ* background [66, 103]. The importance of Smc5/6 in late S/G2 phase, however, does not exclude a role of the complex during replication, underlined in this report by Smc5/6 ability to co-purify with and SUMOylate Pol ε at replication forks.

Extensive analysis of *pol2-K571* or *po2-sim* did not show any robust defect in genome stability maintenance in response to fork stalling. One possible obstacle in elucidating this function might be the redundancy of the pathways regulating replication forks and Pol ε. In fact, Pol2 SUMOylation might just be one of the many targets downstream of the S phase checkpoint response. Since several replisome components are phosphorylated following checkpoint activation, [21, 22, 26, 104]), the loss of a single target of regulation might not produce a phenotype and only removal of such redundant regulation might elucidate the role of this modification. Interestingly, results from the Zhao lab indicate that *pol2 K571R* is synthetic defective with the allele *dpb2-1*, leading to an increase in genomic instability and DNA damage sensitivity (Xiaolan Zhao, personal communication). Since *dpb2-1* shows a weaker association with Pol2 C-terminal [105], this might suggest that, following partial destabilisation of Pol ε, Pol2 SUMOylation becomes critical in promoting/regulating DNA synthesis. In addition, the SUMOylation might regulate the conformation of Pol ε or it’s functional engagement to the rest of the replisome and this could become critical once the interaction with Dpb2 is weakened. Interestingly, analysis by Electron Microscopy shows that the flexible linker between the catalytic N-terminal half of Pol2 and the Pol2 C-terminal / Dpb2 / CMG complex allows either for a compressed conformation of Pol2, or for an extended one [106]. It is intriguing to speculate that, since Pol2 N-terminal is SUMOylated and Pol2 C-terminal contains a SIM, this intramolecular interaction might regulate the different states of conformation of Pol2. This could represent a mechanism that helps regulate Pol ε activity in response to stalling, thus promoting fork restart once the obstacles are removed. Importantly, Rad53 actively regulates replisome progression in response to checkpoint activation [21, 27–29] and the SUMOylation of Pol ε might form part of this regulation. Alternatively, *in vivo*, this interaction might promote Pol ε dimerization at replication factories [107] or regulate the catalytic activity of the protein. Interestingly, the idea of intramolecular interaction between the N-terminal and the C-terminal of Pol2 or its dimerization is supported by the observation that the two halves of Pol2 strongly interact even after the cleavage of the flexible region connecting them (S8A and S8B Fig). While we didn’t observed large changes in the level of interaction between Pol2 N-terminal and C-terminal between the SUMOylated and the non-SUMOylated form of the protein, more sensitive approaches will be required for testing the possibility. The use of genetic code expansion and chemical biology approaches [108], might provide sufficient amounts of specifically SUMOylated Pol ε and test this model.

Alternatively, K571-SUMO the Pol2-SIM, either independently or in a co-ordinated manner, might be required at the sites of DNA synthesis stalling for the effective recruitment of factors required for the protection or repair of replication forks (model shown in Fig 6E). Although the presence or absence of SUMOylation did not qualitatively affect the pattern of proteins co-immunoprecipitating with Pol ε, it would interesting to analyse whether the levels of recruitment and the strength of some of these interactions is affected in *pol2 K571R* and *pol2-sim* mutants.

All together, here we have described a novel Rad53-dependent mechanism of regulation of the replisome mediated by the Smc5/6 complex. While the role in genome stability of the SUMO-ligase activity of Mms21 in higher eukaryotes is still poorly understood [40, 62, 109], the high conservation of the Smc5/6 complex, Pol ε and the S phase checkpoint mean that it will be of great interest to explore whether Pol2 SUMOylation also regulates replisome function in response to replication stress in other species.

## Material and methods

### Yeast strains and growth conditions

The yeast strains used in this study are isogenic to strain W303-1 (*ade2-1 ura3-1 his3-11*,*15 trp1-1 leu2-3*,*112 can1-100*), unless stated otherwise. The strains used are listed in S1 Table.

All yeasts were grown in YP medium (1% yeast extract, 2% peptone) supplemented with glucose (YPD), galactose (YPGal), or raffinose (YPRaf) to a final concentration of 2% (w/v). For solid media, the same recipe was used, but with a final concentration of 1% (w/v) agar. Alternatively, minimal SC medium was used (ammonium sulphate 0.5% w/v, yeast Nitrogen base 0.17% w/v, glucose 2%, SC mix 0.139% (Sigma-Aldrich Y2021), agar 1%), supplemented with the required amino acids. The default temperature used in the experiments is 24˚C, unless specifically indicated in the experiment. For cell cycle experiment, cells were grown to exponential phase (0.7 x 10^7^ cells/ml) synchronized in G1 by addition of 7.5 μg/ml α-factor mating pheromone (Pepceuticals) and released into S phase by washing twice with fresh YPD media. To induce replication stress, hydroxyurea (HU; Molekula) was added to a final concentration of 0.2 M, methyl methane sulphonate (MMS, Sigma) to a final concentration of 0.033% v/v, or Camptothecin (CPT, Sigma) to a final concentration of 20 μM. Cells were arrested in G2-phase by addition of 5 μg/ml nocodazole (Sigma-Aldrich M1404) to the culture medium for one generation time; double stranded breaks were induced with Zeocin (ZEO, Gibco) to a final concentration of 70 μM. For analysis asynchronous cultures, cells were grown to the concentration of 1 x 10^7^ cells/ml.

Yeast-Two-Hybrid analysis based on the Gal4 transcription factor was performed by co-transformation of derivatives of pGADT7 (Gal4-activation domain-HA tag (LEU2); Clontech) or pGBKT7 (Gal4-DNA binding domain-MYC tag (TRP1); Clontech) into the yeast strains PJ69–4A. SC medium was used, either lacking Tryptophan and Leucine (selective for pGADT7 and pGBKT7, but non-selective for the two-hybrid interaction) or lacking Tryptophan, Leucine, Histidine or Tryptophan, Leucine, Histidine, Adenine (weak and strong selection for the two-hybrid interaction, respectively).

For yeast two-hybrids experiments, cells were grown on selective media, until single colonies were visible. From each strain, five discrete colonies of similar size were resuspended in sterile deionised water, counted, and diluted to the appropriate concentration. From this suspension, serial dilutions (5 x10^6^, 5 x10^5^, 5 x10^4^, 5 x10^3^ cells/ml) were generated. Finally, 10 μl of the solutions were plated.

### Generation of mutants

Gene deletions and tagging were made by one step PCR transformation in diploids, followed by sporulation and tetrad dissection as in [110, 111]. The allele *pol2-3xTEV-9MYC* was generated by first inserting the URA3 gene between 3681bp - 3727bp of POL2 ORF. Cells were then transformed with a construct generated by fusion PCR and composed of 3127–3681 (*POL2* ORF)-3xTEV sequence- 3682–6666 (*POL2* ORF)- 9MYC tag- *K*.*l*. *TRP1*- +81–518 (downstream *POL2* ORF). The 3xTEV sequence inserted is GASENLYFQGATASENLYFQGSATGAENLYFQGAG. The fragment was transformed and selected first with *K*.*l*.*TRP*1 and then for loss of the *URA3* marker. For selection of *ura3* cells, 5-Fluoroorotic acid (5-FOA; F5001, Melford Laboratories) was added to a final concentration of 1% w/v in SC medium. Positive clones were analysed by PCR, immunoblotting and sequencing. The allele *pol2K571R* was generated with a similar strategy, first by deleting the region -95bp to +1726bp of *POL2* ORF by insertion of a *URA3* allele, then by generating by fusion PCR a construct -145bp to -95bp (upstream *POL2* ORF) -*K*.*l*. *TRP1*- with– 370bp to + 2049bp (1712 AGA 1714 to gaA ORF POL2). Cells were first selected in SC-TRP medium, followed by SC 5-FOA. Positive clones were analysed by PCR and sequencing. The allele *pol2sim* was generated by fusion PCR using a strategy similar to C-terminal tagging, but keeping the stop codon at the end of the ORF so to avoid introducing any tag at the end of the protein. This introduces the mutation between position 6627bp– 6643bp (POL2 ORF) from TTTGATATATTATTG to gcTGATgcAgccgct. Positive cells were tested by PCR and sequencing.

### Plasmids

The lists of the plasmids used in this study are in Appendix Supplementary S2 Table. Two-hybrid plasmids were generated by recombination in budding yeast, by co-transforming linearized versions of pGADT7 or pGBKT7 (digested with NdeI-XhoI and NdeI-PstI, respectively) into yeast cells, together with PCR products that contained the test sequence flanked by 50 bp homology to the digested vector. Positive clones were selected in SC-Leucine and SC- Tryptophan (for pGADT7 and pGBKT7, respectively). The recombined plasmids were then recovered from yeast, amplified and sequenced. Point mutations of CysA, CysB and the SIM were introduced by fusion PCR using synthetic DNA as a template (Biomatik).

### Protein analysis

Immunoprecipitation of replication proteins were conducted as previously described [21, 112]. For immunoblotting experiments, about 2.5 × 10^9^ cells were used, while for mass spectrometry analysis in Fig 2A and 2B (Sld5 IP) and 5B, about 1× 10^10^ cells were used. For each sample, 2.5 g of cells was ground in a SPEX SamplePrep 6780 Freezer/Mill. For Fig 2B (Sld5 IP), the eluted material was also precipitated with ice cold 20% TCA and resuspended in 1x Laemmli buffer supplemented with 150mM Tris. For protein dephosphorylation, immunoprecipitated material was washed three times before being washed once with the reaction buffer (as provided by the manufacturer) and then resuspended in a final volume of 50 μl and incubated with 400 units of lambda phosphatase (New England Biolabs). A mock-treated control and a sample treated with lambda phosphatase and also the phosphatase inhibitors—20 mM NaVO_4_, and 50 mM NaF—were also included in the experiment as controls. Protein samples were incubated at 30˚C for 30 min. Samples were then boiled in Laemmli buffer and eluted. For the cleavage with TEV, samples were resuspended in wash buffer 100 mM potassium acetate without protease inhibitors and incubated with 20 units of AcTEV (Invitrogen) and incubated for 2h at 24˚C. Analysis of the signal intensity was conducted using Fiji. Unsaturated exposures were used for the analysis. Cross-linking immunoprecipitations were conducted as in [21].

Trichloroacetic acid (TCA) protein extraction was conducted as described before [113]. The TAP tag was detected using Peroxidase:Anti-Peroxidase complex (Sigma‐Aldrich). Other proteins were detected by immunoblotting using polyclonal antibodies previously described [21, 114, 115], polyclonal anti-FLAG antibody (Sigma‐Aldrich), 9E10 anti-MYC antibody (Biorad), polyclonal anti-Rad53 antibody (Santa Cruz) monoclonal anti-Ubiquitin (P4D1) and polyclonal antibody specific for a histone H2A and histone H2A phosphorylated at Serine 129 (Abcam). The anti-SUMO antibodies used were the described in [116] or raised in sheep using the full-length Smt3 protein and purified using His_6_-Smt3GGΔ.

### MS and analysis

For mass spectrometry analysis in Fig S2, each sample lane was run into pre-cast Novex Wedgewell 10% Tris-Glycine polyacrylamide gels and ran in the supplied MOPS buffer for 10mm then cut into 10 bands before digestion with trypsin. Peptides were analysed by nanoliquid chromatography–tandem mass spectrometry with an Orbitrap Fusion (Proteomics Research Technology Platform, University of Warwick). For mapping of the SUMOylation site, analysis of the trypsin-digested peptides was conducted by MaxQuant.

### SUMOylation *in vitro*

His_6_-Smt3GGΔ, Ubc9-His_6_, and His_6_-Aos1/Uba2-His_6_ (co-expressed in the same cell) were expressed in *E*. *coli* (Rosetta) and purified by Ni-NTA affinity purification as previously described in [117, 118]. Smt3GGΔ and Ubc9 preparations were >99% pure and the Uba2/Aos1 preparation >90%. All proteins were dialyzed (Slide-A-Lyzer Dialysis Cassettes, 10K MWCO, Pierce) with a buffer 10% Glycerol, 50 mM HEPES (pH 7.0), 100 mM NaCl, 10 mM MgCl_2_, 0.1 mM DTT, 5 mM Tris, 20 mM imidazole, 0.5 μM ZnCl_2_. Yeast proteins were purified from 5g (TAP-Dpb2) of 2.5g (Smc5-TAP) cell pellet as described above. The last couples of washes of the magnetic beads were performed in SUMOylation buffer (without ZnCl_2_). The *in vitro* reactions were performed following AcTEV elution of TAP-Smc5 and TAP-Dpb2. Reactions were performed in siliconised low retention 1.5 tubes and contained 50 mM HEPES (pH 7.0), 100 mM NaCl, 10 mM MgCl_2_, 0.1 mM DTT, 20 μg/ml bovine serum albumin (BSA), 5 mM Tris, 20 mM pH 8, imidazole, 0.5 μM ZnCl_2_ and the following as noted: 5 mM ATP; 5mM SUMO; 150nM Uba2/Aos1 and 150 nM Ubc9, Pol2 (20 μl of TEV eluted material) and Smc5 (5-10-15 vl of final elute). Reactions were conducted at 30˚C for 1h. Time was started following addition of ATP to the reaction.

### Measurement of DNA content

Cells were fixed and prepared for flow cytometry as described previously [119] and then analysed with a FACScan flow cytometer (Becton-Dickinson) and BD CellQuest (BD Biosciences).

## Supporting information

S1 Fig Pol2 SUMOylation is mainly stimulated by HU. **A)** Pol2 modification is not sensitive to phosphatase treatment. Cells carrying a TAP-tagged version of DPB2 were grown to exponential phase, arrested in G1 and synchronously released in S phase for 30 min in YPD (S phase) or for 90 min in YPD 0.2 M HU (HU). Pol ε was purified under stringent conditions (700 mM potassium acetate) and incubated with lambda phosphatase, both in the presence or absence of the phosphatase inhibitors 20 mM sodium vanadate (NaVO_4_) and 50 mM sodium fluoride (NaF). A mock-treated sample was used as a control. While the phosphatase is able to dephosphorylate components of the replisome (such as Mcm4), the upper band appearing over Pol2 in HU is not affected. **B)** Pol2 is not ubiquitylated. Samples from Fig 1B were probed by immunoblotting with anti-Ubiquitin antibody. No specific band is recognised in the Dbp2-TAP immunoprecipitated material following HU exposure. **C)** Pol2 is SUMOylated preferentially in response to HU treatment. Cells carrying a TAP-tagged version of Dpb2, as well a tagged version of Sld3 and Mrc1, were treated as described in Fig 1A. Dpb2 was then purified and analysed by immunoblotting. Pol2 SUMOylation occurs to a low level during a normal phase and it is preferentially stimulated following HU treatment. **D)** Analysis of checkpoint activation following different genotoxic treatments. Cells from the experiment above were collected; protein samples were obtained by TCA extraction and analysed by immunoblotting. Strikingly, MMS stimulates the phosphorylation of Mrc1, Sld3 and Rad53 to a similar extent than HU.
(TIF)

S2 Fig Mass spectrometry analysis of the replicative DNA polymerases. The samples shown in Fig 2A were analysed by mass spectrometry. We observe that all the subunits of the DNA polymerases are present.
(TIF)

S3 Fig Defects in Rad53 activation greatly decrease Pol2 SUMOylation in HU. A) Wild type, *ctf18Δ* and *ctf18-2A* (76) cells were arrested in G1 and synchronously released in YPD 0.2 M HU. Cells samples were taken every 15 min and analysed by immunoblotting. B) The S phase checkpoint is required for the 10-fold increase in Pol2 SUMOylation in response to HU. Wild type, *mrc1Δ* and *rad53Δ*, all deleted for *SML1* and carrying a TAP-tagged allele of *DPB2*, were arrested in G1 before being released in the medium containing 0.2 M HU or 0.03% MMS for 90 min. Celle extracts were used for immunoprecipitation of TAP and analysed by immunoblotting.
(TIF)

S4 Fig Analysis of the sensitivity to auxin- of the mutants *mms21-aid*, *smc5-aid* and *scc2-aid scc4-aid*. **A)** Serial dilutions were spotted on YPGal plates, with or without 0.5 mM IAA, and incubated at 30˚C for 2 days. **B)** FACS analysis of the cell cycle progression following depletion of Smc5, Mms21 and Scc2 Scc4. Cells were grown in YPRaf to the exponential phase, arrested in G1, resuspended in YPGal for 35 min, incubated for 60 min in medium containing a final concentration of 0.5 mM IAA to induce protein degradation, and released in YPGal 0.2 M HU and 0.5 mM IAA for 90 min. At this point, the samples used for the experiment show in Fig 4B were taken. Cells were then washed twice and released in YPGal 0.5 mM IAA. α-factor was added to block re-entry in the next cell cycle.
(TIF)

S5 Fig Pol2 SUMOylation depends on Mms21. **A)** Mms21 is required for the basal levels of SUMOylation during S phase. Cells were treated as in Fig 4A, but released in S phase in YPD medium for 30 min. Dpb2-TAP was immunoprecipitated and protein samples were analysed by immunoblotting. **B)** Rad53 does not regulate the basal levels of SUMOylation in S phase. Wild type, *rad53Δ* and *rmm3Δ* cells, all deleted for *SML1* and carrying a TAP-tagged allele of *DPB2*, were synchronously released in S phase in YPD medium for 30 min at 24˚C. Dpb2-TAP was immunoprecipitated and protein samples were analysed by immunoblotting. **C)** Point mutation of the sp-RING domain of *MMS21* blocks Pol2 SUMOylation. Cells carrying a TAP-tagged version of Dpb2 and a *Mms21* or *mms21-(C200A H202A) (mms21-CH)* allele were grown to the exponential phase, arrested in G1, and released in YPD 0.2 M HU. Dpb2-TAP was immunoprecipitated and analysed by immunoblotting. **D)** Re-expression of Mms21 leads to Pol2 SUMOylation. Cells Dpb2-TAP *mms21-CH*, with or without a second allele of *MMS21* under the *GAL1*,*10* promoter, were grown in YPRaf to the exponential phase, arrested in G1, and synchronously in YPRaf medium containing 0.2 M HU for 75 min. Cells were resuspended and incubated for further 60 min either in YPRaf 0.2 M HU or YPGal 0.2 M HU. Dpb2-TAP was immunoprecipitated and analysed by immunoblotting. Mms21 re-expression leads to the SUMOylation of Pol2 (its co-immunoprecipitation with Dpb2).
(TIF)

S6 Fig Pol ε and the Smc5/6 complex interact both in G1 and S phase, independently of the S phase checkpoint. **A)** Mms21 co-immunoprecipitates with Dpb2. Cells were treated as in Fig 4E, but instead of a FLAG-tagged version of Smc5, they carried a FLAG-tagged version of Mms21. **B)** Pol ε co-immunoprecipitates with Smc5. Cells carrying either a TAP-tagged or untagged version of Smc5 were grown to exponential phase, arrested in G1, and released either in YPD for 30 min (S) or in YPD 0.2 M HU for 90 min (HU). Smc5-TAP was immunoprecipitated and protein samples were analysed by immunoblotting. **C)** Analysis of the salt-sensitivity of the interaction between Dpb2 and Mms21. Cells carrying a TAP-tagged version of Dpb2 and a FLAG-tagged version of Mms21 were grown to exponential phase, arrested in G1, and released in YPD for 30 min. Dpb2-TAP was immunoprecipitated and washed in solutions containing different concentration of potassium acetate (KOAc), as indicated in the figure. The IPs were eluted by boiling in Laemmli buffer and protein samples were analysed by immunoblotting. **D)** Mec1 and the SUMO-ligase activity of Mms21 are not required for the co-purification between Smc5 and Dpb2. Cells *sml1Δ*, *sml1Δ mec1Δ*, *sml1Δ rad53Δ*, *mrc1Δ* and *mms21-CH* carrying either a tagged version of *DPB2* were grown to exponential phase, arrested in G1, and released either in YPD for 30 min (S) or in YPD 0.2 M HU for 90 min (HU). Dpb2-TAP was immunoprecipitated and protein samples were analysed by immunoblotting
(TIF)

S7 Fig Pol2 C-terminal interacts with SUMO. **A)** Controls for the interactions shown in Fig 6A. The indicated fragments were transformed either with an empty plasmid (negative control) and an interacting fragment (positive control). **B)** Controls for the interactions shown in Fig 6B. The indicated fragments were transformed either with an empty plasmid (negative control, top) or with a plasmid expressing Dpb2 (positive control, bottom). **C)** The conserved CysA and CysB sequences at Pol2 C-terminal do not mediate the interaction with SUMO. Wild type Pol2 fragment 2103–2222, an allele mutated for CysA (*C2108S C2111S*, *CysA_mut*), and an allele mutated for CysB (*C2164S C2167S*, *CysB_mut*) were tested for the ability to interact with SUMO (Smt3AAΔ). Negative (empty plasmid) and positive controls (Dpb2) were also included. As previously reported (103), CysB plays a major role in mediating the interaction with Dpb2.
(TIF)

S8 Fig Pol2 N-terminal and C-terminal halves interact. **A)** Wild type and *mms21-CH* cells, carrying the *POL2-(3TEV)-9MYC* allele at the genomic locus, were grown to exponential phase, arrested in G1 and synchronously released in YPD 0.2 M HU for 90 min. Pol2 was then immunoprecipitated with anti-MYC beads under high salt conditions (500 mM potassium acetate) before incubation with TEV protease at 24˚C for 2 h. Next, sample were eluted by boiling (no wash), or washed five times with buffers with different salt concentration (100 mM, 300 mM or 1M potassium acetate), before elution by boiling. All samples were analysed by immunoblotting. The N-terminal part of Pol2 was detected with a polyclonal antibody raised against the N-terminal half of Pol2, while the C-terminal half was examined with an anti-MYC antibody. The asterisk indicates a background band. **B)** Analysis of the signal in **A)**. The plots show the ratio of the signal of the N-terminal and SUMO immunoblots divided by the signal of the C-terminal blot. Both the cut and full-length (FL) fractions were analysed. The ratios were then normalized against the values of “no wash”.
(TIF)

S1 Table List of yeast strains. (DOCX)

S2 Table List of plasmids. (DOCX)
