# Supplementary figures and images for: The S phase checkpoint promotes the Smc5/6 complex dependent SUMOylation of Pol2, the catalytic subunit of DNA polymerase ε
Source: PLoS Genet. 2019 Nov 25;15(11):e1008427. doi: 10.1371/journal.pgen.1008427 (PMC6876773; doi:10.1371/journal.pgen.1008427)

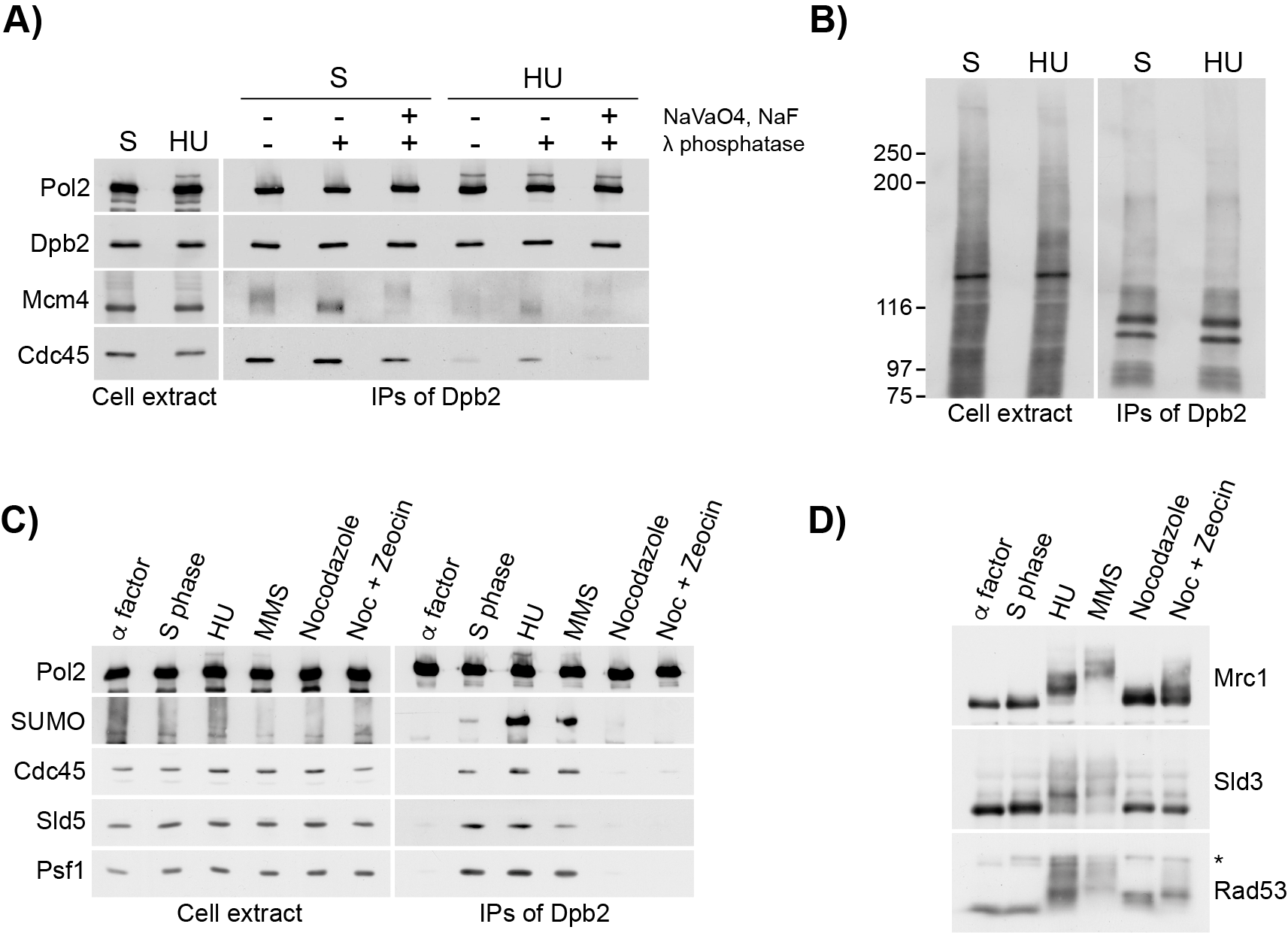

Supplement: S1 Fig — A) Pol2 modification is not sensitive to phosphatase treatment. Cells carrying a TAP-tagged version of DPB2 were grown to exponential phase, arrested in G1 and synchronously released in S phase for 30 min in YPD (S phase) or for 90 min in YPD 0.2 M HU (HU). Pol ε was purified under stringent conditions (700 mM potassium acetate) and incubated with lambda phosphatase, both in the presence or absence of the phosphatase inhibitors 20 mM sodium vanadate (NaVO4) and 50 mM sodium fluoride (NaF). A mock-treated sample was used as a control. While the phosphatase is able to dephosphorylate components of the replisome (such as Mcm4), the upper band appearing over Pol2 in HU is not affected. B) Pol2 is not ubiquitylated. Samples from Fig 1B were probed by immunoblotting with anti-Ubiquitin antibody. No specific band is recognised in the Dbp2-TAP immunoprecipitated material following HU exposure. C) Pol2 is SUMOylated preferentially in response to HU treatment. Cells carrying a TAP-tagged version of Dpb2, as well a tagged version of Sld3 and Mrc1, were treated as described in Fig 1A. Dpb2 was then purified and analysed by immunoblotting. Pol2 SUMOylation occurs to a low level during a normal phase and it is preferentially stimulated following HU treatment. D) Analysis of checkpoint activation following different genotoxic treatments. Cells from the experiment above were collected; protein samples were obtained by TCA extraction and analysed by immunoblotting. Strikingly, MMS stimulates the phosphorylation of Mrc1, Sld3 and Rad53 to a similar extent than HU. (TIF) [file pgen.1008427.s001.tif]

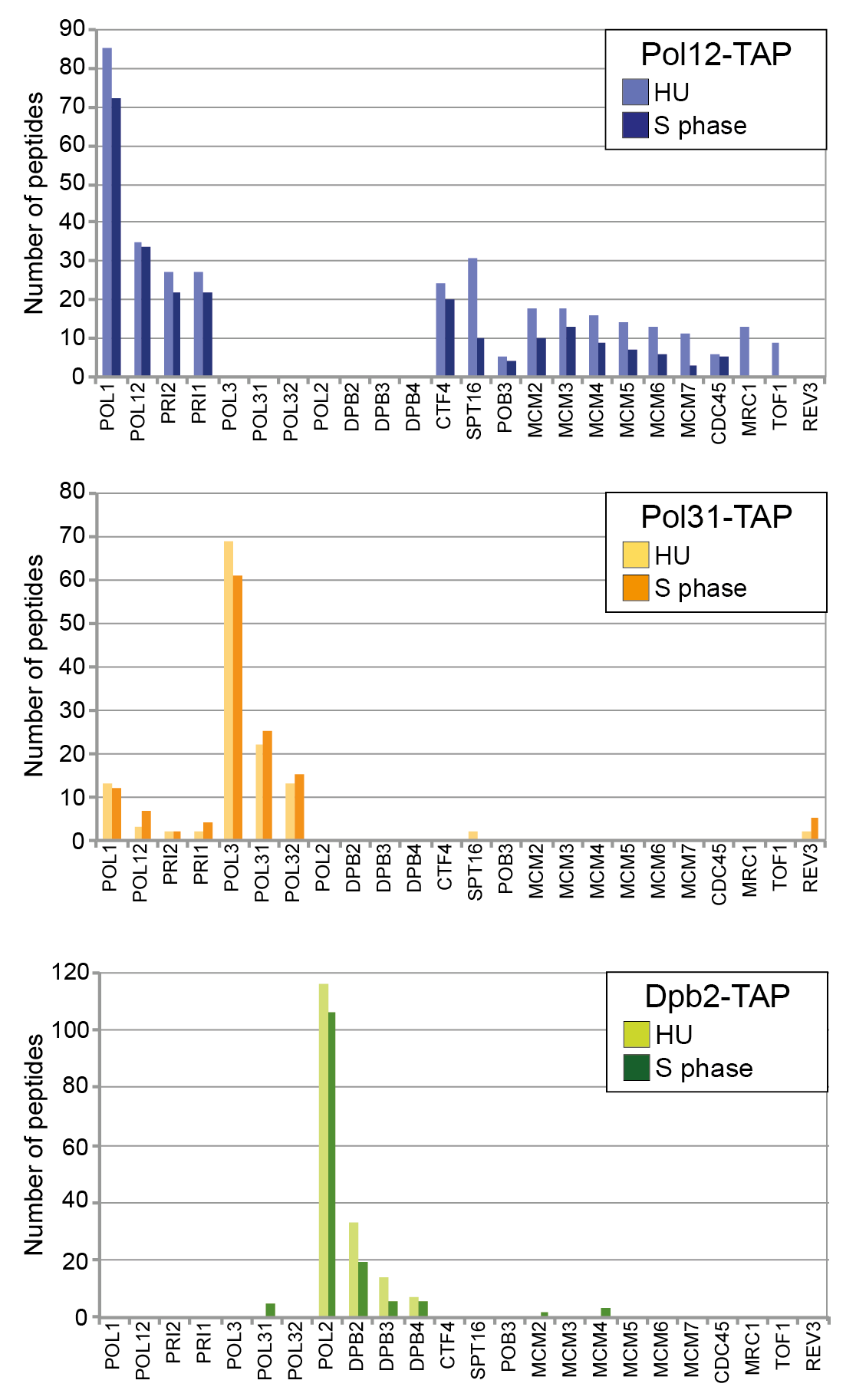

Supplement: S2 Fig — The samples shown in Fig 2A were analysed by mass spectrometry. We observe that all the subunits of the DNA polymerases are present. (TIF) [file pgen.1008427.s002.tif]

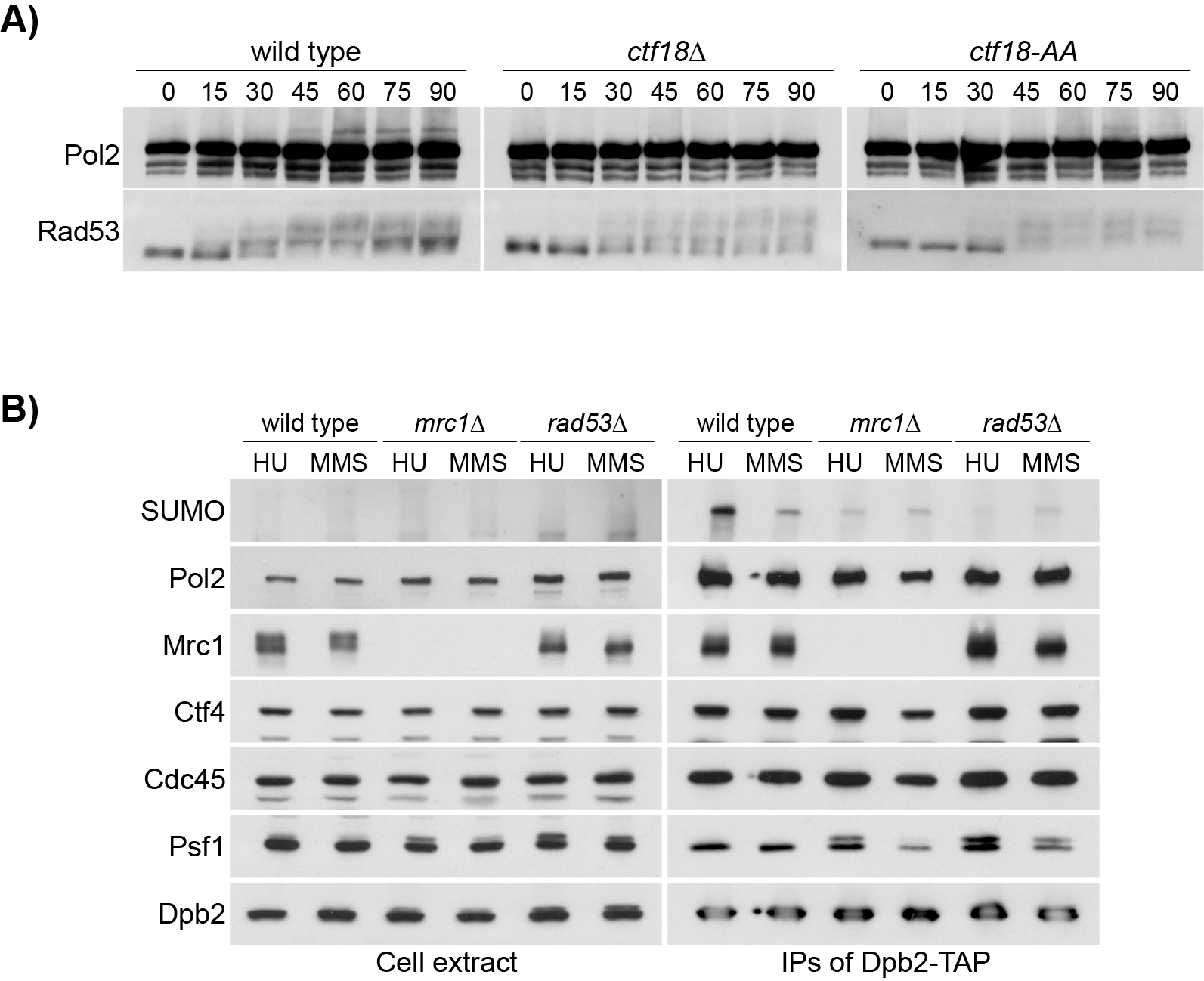

Supplement: S3 Fig — A) Wild type, ctf18Δ and ctf18-2A (76) cells were arrested in G1 and synchronously released in YPD 0.2 M HU. Cells samples were taken every 15 min and analysed by immunoblotting. B) The S phase checkpoint is required for the 10-fold increase in Pol2 SUMOylation in response to HU. Wild type, mrc1Δ and rad53Δ, all deleted for SML1 and carrying a TAP-tagged allele of DPB2, were arrested in G1 before being released in the medium containing 0.2 M HU or 0.03% MMS for 90 min. Celle extracts were used for immunoprecipitation of TAP and analysed by immunoblotting. (TIF) [file pgen.1008427.s003.tif]

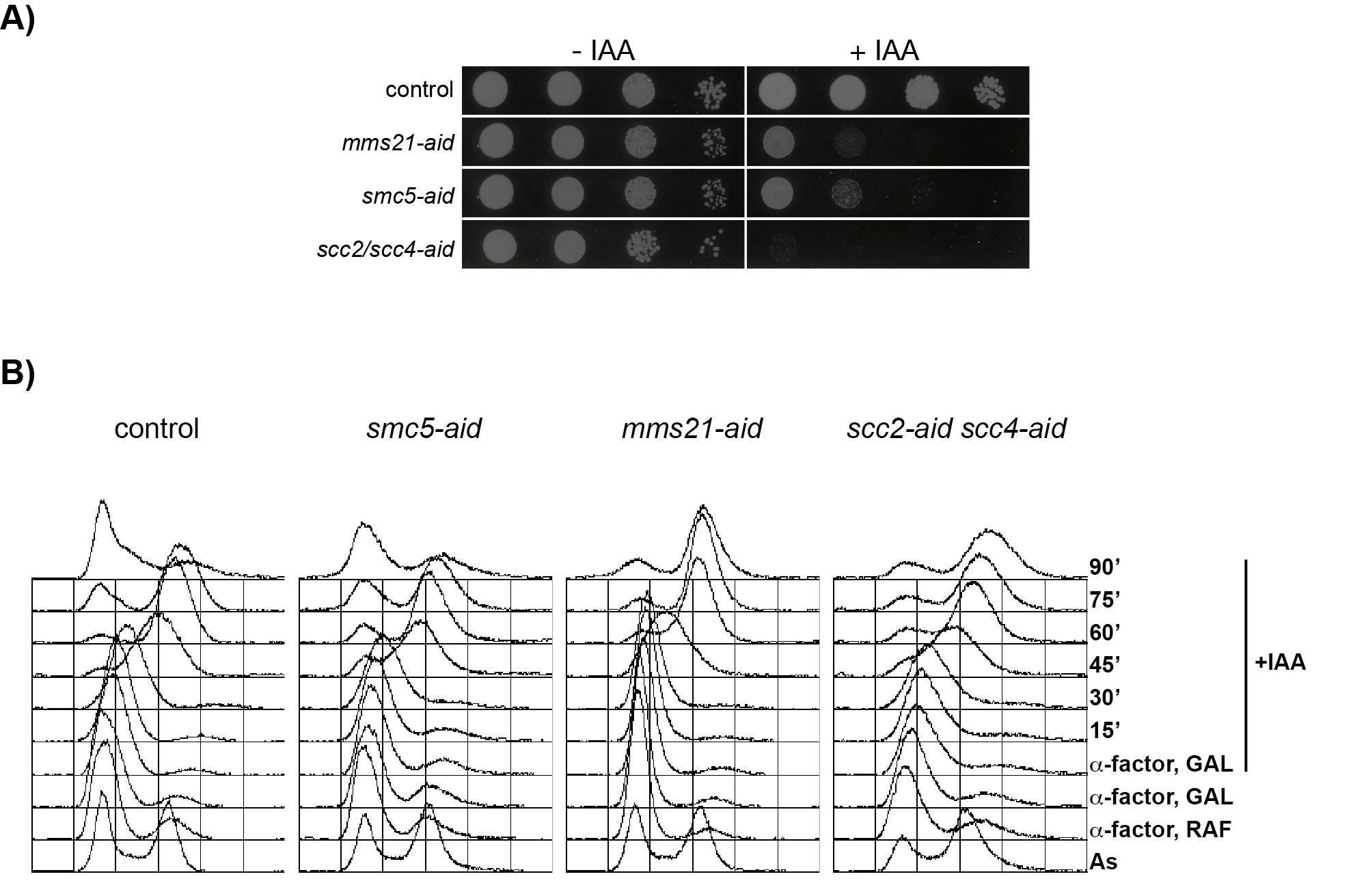

Supplement: S4 Fig — A) Serial dilutions were spotted on YPGal plates, with or without 0.5 mM IAA, and incubated at 30˚C for 2 days. B) FACS analysis of the cell cycle progression following depletion of Smc5, Mms21 and Scc2 Scc4. Cells were grown in YPRaf to the exponential phase, arrested in G1, resuspended in YPGal for 35 min, incubated for 60 min in medium containing a final concentration of 0.5 mM IAA to induce protein degradation, and released in YPGal 0.2 M HU and 0.5 mM IAA for 90 min. At this point, the samples used for the experiment show in Fig 4B were taken. Cells were then washed twice and released in YPGal 0.5 mM IAA. α-factor was added to block re-entry in the next cell cycle. (TIF) [file pgen.1008427.s004.tif]

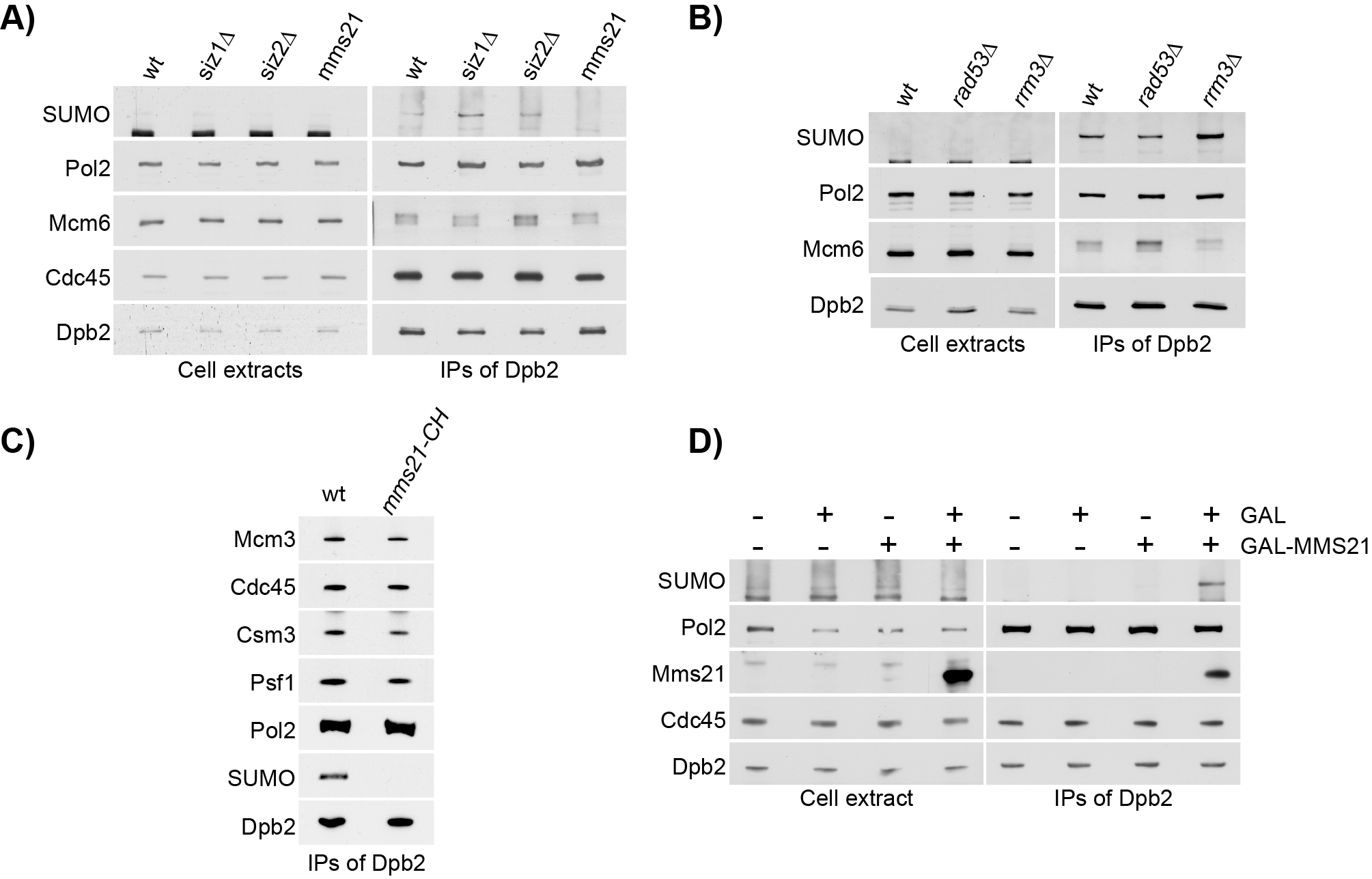

Supplement: S5 Fig — A) Mms21 is required for the basal levels of SUMOylation during S phase. Cells were treated as in Fig 4A, but released in S phase in YPD medium for 30 min. Dpb2-TAP was immunoprecipitated and protein samples were analysed by immunoblotting. B) Rad53 does not regulate the basal levels of SUMOylation in S phase. Wild type, rad53Δ and rmm3Δ cells, all deleted for SML1 and carrying a TAP-tagged allele of DPB2, were synchronously released in S phase in YPD medium for 30 min at 24˚C. Dpb2-TAP was immunoprecipitated and protein samples were analysed by immunoblotting. C) Point mutation of the sp-RING domain of MMS21 blocks Pol2 SUMOylation. Cells carrying a TAP-tagged version of Dpb2 and a Mms21 or mms21-(C200A H202A) (mms21-CH) allele were grown to the exponential phase, arrested in G1, and released in YPD 0.2 M HU. Dpb2-TAP was immunoprecipitated and analysed by immunoblotting. D) Re-expression of Mms21 leads to Pol2 SUMOylation. Cells Dpb2-TAP mms21-CH, with or without a second allele of MMS21 under the GAL1,10 promoter, were grown in YPRaf to the exponential phase, arrested in G1, and synchronously in YPRaf medium containing 0.2 M HU for 75 min. Cells were resuspended and incubated for further 60 min either in YPRaf 0.2 M HU or YPGal 0.2 M HU. Dpb2-TAP was immunoprecipitated and analysed by immunoblotting. Mms21 re-expression leads to the SUMOylation of Pol2 (its co-immunoprecipitation with Dpb2). (TIF) [file pgen.1008427.s005.tif]

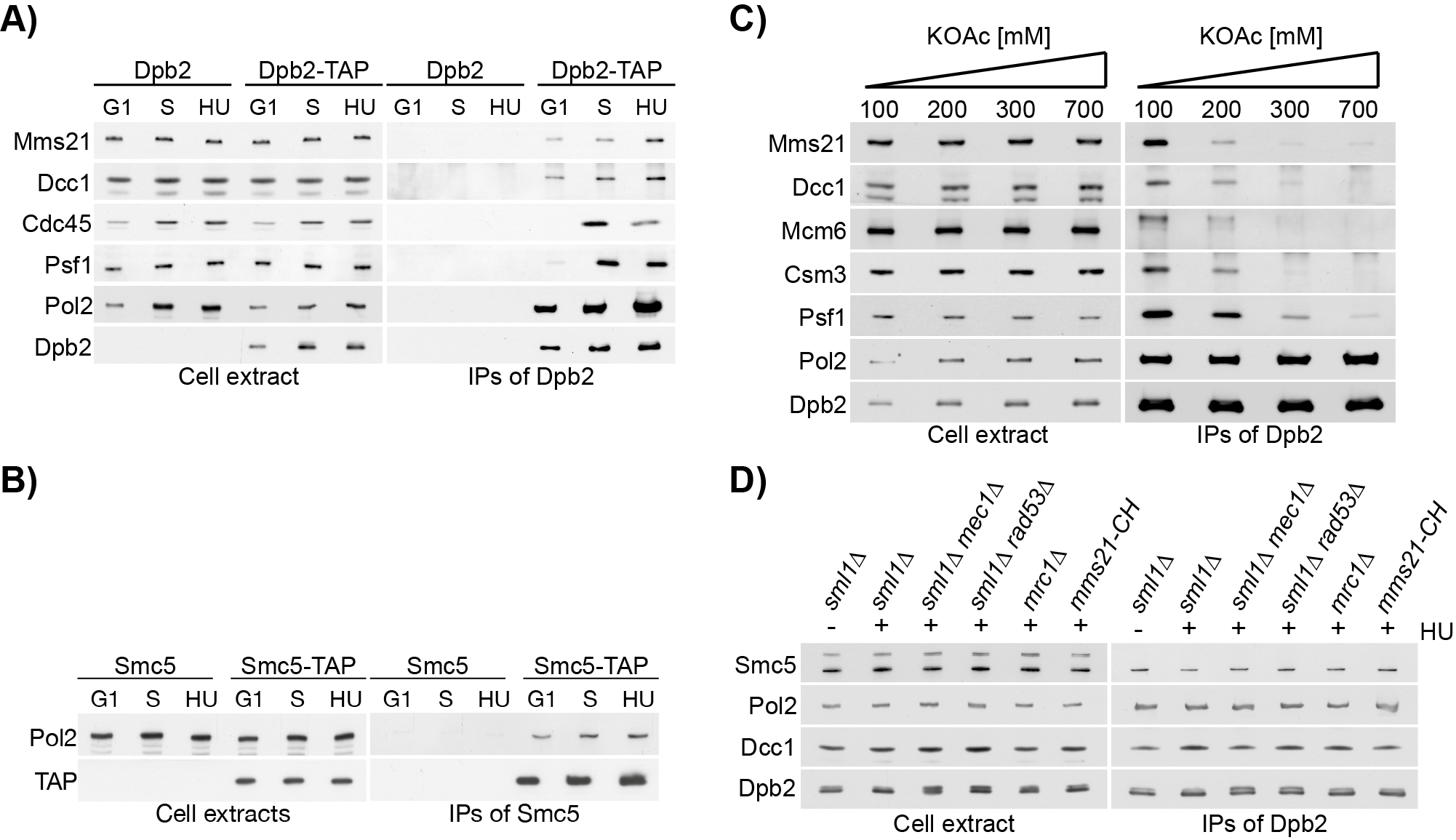

Supplement: S6 Fig — A) Mms21 co-immunoprecipitates with Dpb2. Cells were treated as in Fig 4E, but instead of a FLAG-tagged version of Smc5, they carried a FLAG-tagged version of Mms21. B) Pol ε co-immunoprecipitates with Smc5. Cells carrying either a TAP-tagged or untagged version of Smc5 were grown to exponential phase, arrested in G1, and released either in YPD for 30 min (S) or in YPD 0.2 M HU for 90 min (HU). Smc5-TAP was immunoprecipitated and protein samples were analysed by immunoblotting. C) Analysis of the salt-sensitivity of the interaction between Dpb2 and Mms21. Cells carrying a TAP-tagged version of Dpb2 and a FLAG-tagged version of Mms21 were grown to exponential phase, arrested in G1, and released in YPD for 30 min. Dpb2-TAP was immunoprecipitated and washed in solutions containing different concentration of potassium acetate (KOAc), as indicated in the figure. The IPs were eluted by boiling in Laemmli buffer and protein samples were analysed by immunoblotting. D) Mec1 and the SUMO-ligase activity of Mms21 are not required for the co-purification between Smc5 and Dpb2. Cells sml1Δ, sml1Δ mec1Δ, sml1Δ rad53Δ, mrc1Δ and mms21-CH carrying either a tagged version of DPB2 were grown to exponential phase, arrested in G1, and released either in YPD for 30 min (S) or in YPD 0.2 M HU for 90 min (HU). Dpb2-TAP was immunoprecipitated and protein samples were analysed by immunoblotting (TIF) [file pgen.1008427.s006.tif]

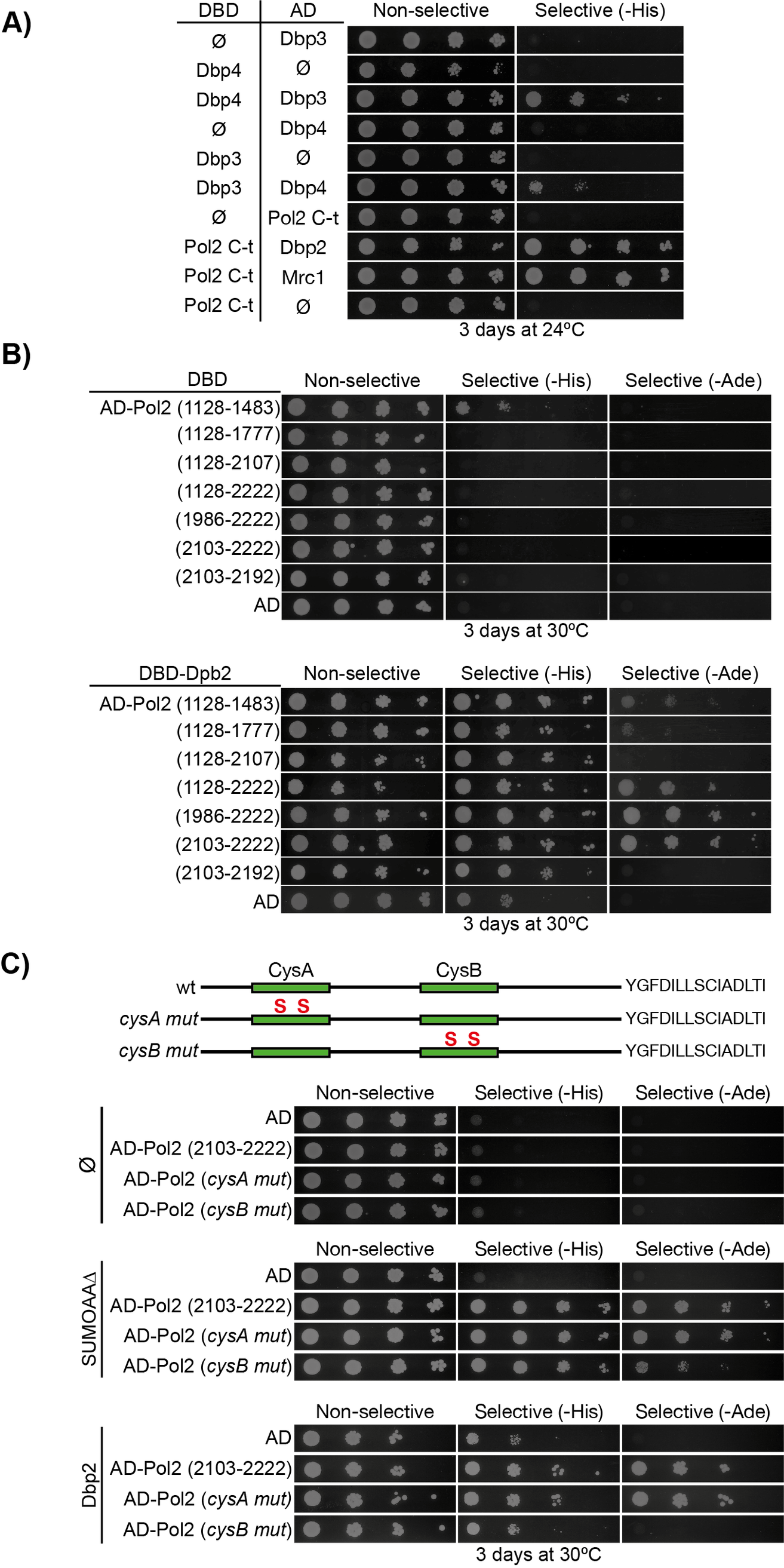

Supplement: S7 Fig — A) Controls for the interactions shown in Fig 6A. The indicated fragments were transformed either with an empty plasmid (negative control) and an interacting fragment (positive control). B) Controls for the interactions shown in Fig 6B. The indicated fragments were transformed either with an empty plasmid (negative control, top) or with a plasmid expressing Dpb2 (positive control, bottom). C) The conserved CysA and CysB sequences at Pol2 C-terminal do not mediate the interaction with SUMO. Wild type Pol2 fragment 2103–2222, an allele mutated for CysA (C2108S C2111S, CysA_mut), and an allele mutated for CysB (C2164S C2167S, CysB_mut) were tested for the ability to interact with SUMO (Smt3AAΔ). Negative (empty plasmid) and positive controls (Dpb2) were also included. As previously reported (103), CysB plays a major role in mediating the interaction with Dpb2. (TIF) [file pgen.1008427.s007.tif]

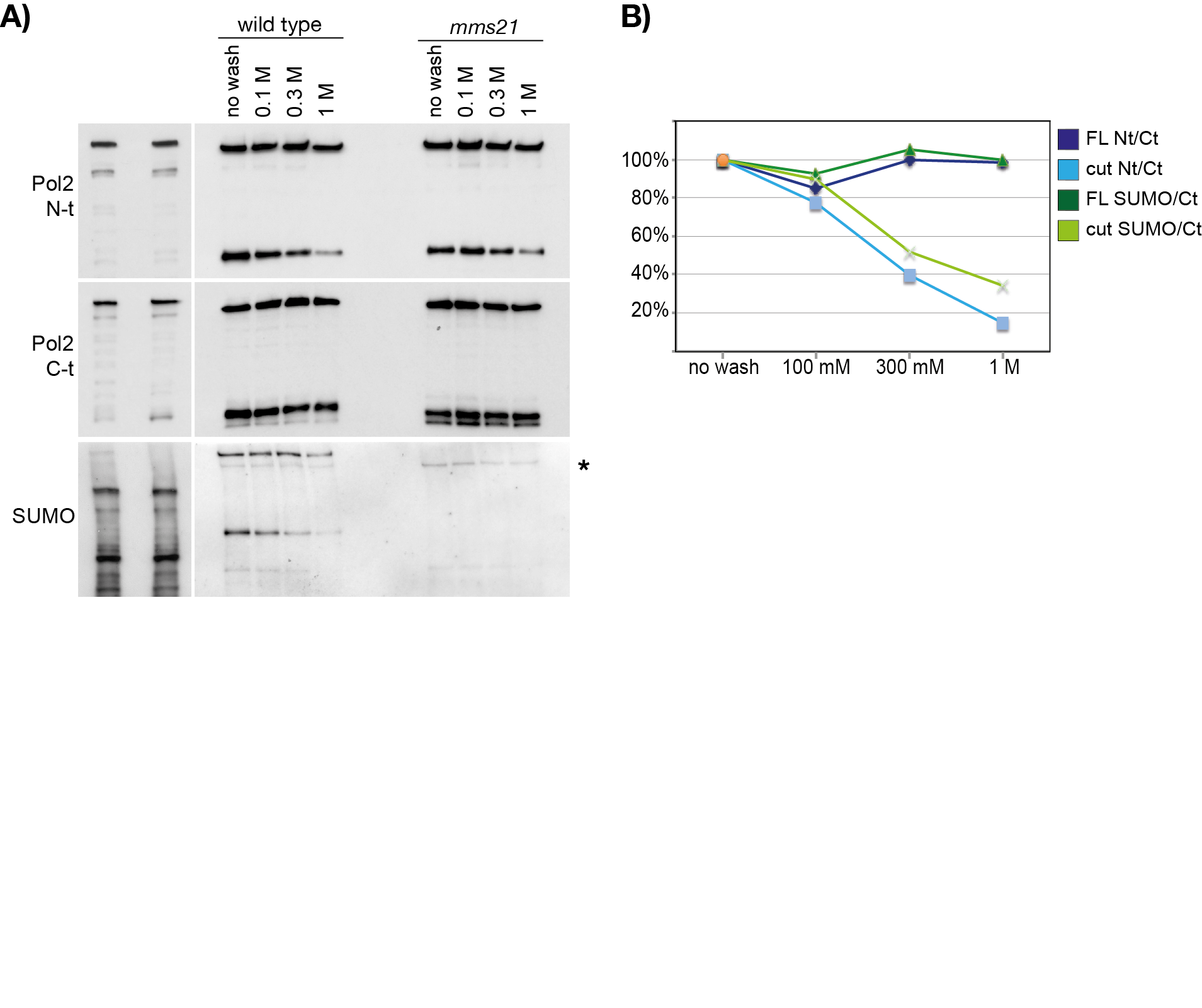

Supplement: S8 Fig — A) Wild type and mms21-CH cells, carrying the POL2-(3TEV)-9MYC allele at the genomic locus, were grown to exponential phase, arrested in G1 and synchronously released in YPD 0.2 M HU for 90 min. Pol2 was then immunoprecipitated with anti-MYC beads under high salt conditions (500 mM potassium acetate) before incubation with TEV protease at 24˚C for 2 h. Next, sample were eluted by boiling (no wash), or washed five times with buffers with different salt concentration (100 mM, 300 mM or 1M potassium acetate), before elution by boiling. All samples were analysed by immunoblotting. The N-terminal part of Pol2 was detected with a polyclonal antibody raised against the N-terminal half of Pol2, while the C-terminal half was examined with an anti-MYC antibody. The asterisk indicates a background band. B) Analysis of the signal in A). The plots show the ratio of the signal of the N-terminal and SUMO immunoblots divided by the signal of the C-terminal blot. Both the cut and full-length (FL) fractions were analysed. The ratios were then normalized against the values of “no wash”. (TIF) [file pgen.1008427.s008.tif]
