# Supplementary material for: The S phase checkpoint promotes the Smc5/6 complex dependent SUMOylation of Pol2, the catalytic subunit of DNA polymerase ε
Source: PLoS Genet. 2019 Nov 25;15(11):e1008427. doi: 10.1371/journal.pgen.1008427 (PMC6876773; doi:10.1371/journal.pgen.1008427)
Supplement: S1 Table — (DOCX) [file pgen.1008427.s009.docx]

Table S1 Yeast strains list

| W303-1 | R. Rothstein | *MATa ade2-1 ura3-1 his3-11,15 trp1-1 leu2-3,112 can1-100 /*  *MATα ade2-1 ura3-1 his3-11,15 trp1-1 leu2-3,112 can1-100* |
| --- | --- | --- |
| W303-1a | R. Rothstein | *MATa ade2-1 ura3-1 his3-11,15 trp1-1 leu2-3,112 can1-100* |
| YAC53 | K. Labib | *MATa sml1∆::HIS3 rad53∆::ADE2* |
| CS29 | K. Labib | *MATa ctf18∆::K.l.TRP1* |
| CS43 | K. Labib | *MATa NTAP2-SLD5 (kanMX) MCM4-5FLAG (hphNT) pep4∆::URA3*  *ADE2+* |
| CS330 | This study | *MATa DPD2-TAP (kanMX) pep4∆::ADE2* |
| CS346 | This study | *MATa DPB2-TAP (kanMX) siz1∆::URA3CP pep4∆::ADE2* |
| CS347 | This study | *MATa DPB2-TAP (kanMX) siz2∆::hphNT pep4∆::ADE2* |
| CS348 | This study | *MATa DPB2-TAP (kanMX) MMS21-3xaid (hphNT) ura3-1::GAL1-OsTIR1-9MYC (K.l. TRP1) pep4∆::ADE2* |
| CS358 | This study | *MATa DPB2-TAP (kanMX) POL2-5FLAG-9HIS (hphNT) pep4∆::ADE2* |
| CS359 | This study | *MATa DPB2-TAP (kanMX) ura3-1::GAL1-OsTIR1-9MYC (K.l. TRP1)*  *SMC5-3xaid (HIS3MX) pep4∆::ADE2* |
| CS361 | This study | *MATa DPB2-TAP (kanMX) ura3-1::GAL1-OsTIR1-9MYC (K.l. lTRP1)*  *SCC2-3xaid (kanMX) SCC4-3xaid (hphNT) pep4∆::ADE2* |
| CS367 | This study | *MATa DPB2-TAP (kanMX) MMS21-5FLAG (hphNT) sml1∆::HIS3 pep4∆::ADE2* |
| CS369 | This study | *MATa DPB2-TAP (kanMX) SMC5-5FLAG (hphNT) sml1∆::HIS3*  *pep4∆::URA3 rad53∆::ADE2* |
| CS370 | This study | *MATa DPB2-TAP (kanMX), MMS21-5FLAG (hphNT) sml1∆::HIS3, pep4∆::URA3 rad53∆::ADE2* |
| CS379 | This study | *MATa DPB2-TAP (kanMX) pep4∆::ADE2 mms21(C200A H202A) (K.l. TRP1)* |
| CS383 | This study | *MATa SMC5-TAP (kanMX), MRC1-18MYC (K.l. TRP1) pep4∆::URA3* |
| CS385 | This study | *MATa DPB2-TAP (kanMX) MRC1-5FLAG (hphNT) SLD3-9MYC (LEU2) pep4∆::ADE2* |
| CS386 | This study | *MATa DPB2-TAP (kanMX) MMS21-5FLAG (hphNT) sml1∆::HIS3, pep4∆::ADE2 ctf18∆::K.l. TRP1* |
| CS387 | This study | *MATa DPB2-TAP (kanMX) MMS21-5FLAG (hphNT) sml1∆::HIS3 pep4∆::ADE2 dcc1∆::hphNT* |
| CS388 | This study | *MATa DPB2-TAP (kanMX) MMS21-5FLAG (hphNT) sml1∆::HIS3 pep4∆::ADE2 mrc1∆::hphNT* |
| CS389 | This study | *MATa DPB2-TAP (kanMX), MMS21-5FLAG (hphNT), sml1∆::HIS3 pep4∆::ADE2, tof1∆::HIS3MX* |
| CS427 | This study | *MATa SMC5-TAP (kanMX) MRC1-18MYC (klTRP1) pep4∆::URA3 (C200A H202A) (klTRP1)* |
| CS445 | This study | *MATa DPB2-TAP (kanMX) mms21(C200A H202A) (klTRP1) pep4∆::ADE2 ura3-1::GAL-MMS21-5FLAG (URA3)* |
| CS447 | This study | *MATa DPB2-TAP (kanMX) SMC5-5FLAG (hphNT) ctf18∆::klTRP1*  *pep4∆::ADE2* |
| CS448 | This study | *MATa DPB2-TAP (kanMX) SMC5-5FLAG (hphNT) mrc1∆::klTRP1*  *pep4∆::ADE2* |
| CS449 | This study | *MATa DPB2-TAP (kanMX) SMC5-5FLAG (hphNT) mms21(C200A H202A) (klTRP1) pep4∆::ADE2* |
| CS459 | This study | *MATa DPB2-TAP (kanMX) SMC5-5FLAG (hphMX) pep4∆::ADE2*  *sml1∆::HIS3 mec1∆::ADE2* |
| CS460 | This study | *MATa DPB2-TAP (kanMX) MMS21-5FLAG (hphMX) pep4∆::ADE2*  *sml1∆::HIS3 mec1∆::ADE2* |
| CS476 | This study | *MATa DPB2-TAP (kanMX), POL2-SMT3_∆GGATY (hphNT), pep4∆::ADE2* |
| CS513 | This study | *MATa pol2_(3TEV sites N1227)-9MYC (klTRP1) pep4∆::ADE2* |
| CS531 | This study | *MATa pol2_(3TEV sites N1227)-9MYC (klTRP1), mms21 (C200A H202A) (K.l. TRP1), pep4∆::ADE2* |
| CS558 | This study | *MATa DPB2-TAP (kanMX) POL2 (klTRP1) pep4∆::ADE2* |
| CS561 | This study | *MATa DPB2-TAP (kanMX) POL2_K571R (klTRP1) pep4∆::ADE2* |
| CS767 | This study | *MATA REV1-5 FLAG (kanMX)* |
| CS936 | This study | *MAT A DPB2-TAP (kanMX) ura3-1::GAL1-OsTIR1-9MYC (klTRP1) pep4∆::ADE2* |
| CS937 | This study | *MATa DPB2-TAP (kanMX) pep4∆::ADE2 esc2∆::HIS3MX* |
| CS938 | This study | *MAT A REV1-5FLAG (kanMX) mms21 (C200A H202A) (klTRP1)* |
| CS1006 | This study | *MATa POL12-TAP (kanMX) pep4Δ::ADE2* |
| CS1007 | This study | *MATa POL31-TAP (kanMX) pep4Δ::ADE2* |
| CS1177 | K. Labib | *MATa ctf18 (W736A, W740A) (K.l. TRP1)* |
| CS3978 | This study | *MATa DPB2-TAP (kanMX) pep4∆::ADE2 rtt107∆::hphNT* |
| CS3982 | This study | *MATa DPB2-TAP (kanMX) pep4∆::ADE2 pol2sim (K.l. TRP1, kanMX)* |
| GDP4 | K. Labib | *MATa sml1∆::HIS3 mec1∆::ADE2* |
| GDP1341 | This study | *MATa DPB2-TAP (kanMX) pep4∆::ADE2 sml1∆::HIS3MX* |
| GDP1343 | This study | *MATa DPB2-TAP (kanMX) pep4∆::ADE2 sml1∆::HIS3 mec1∆::ADE2* |
| GDP1345 | This study | *MATa DPB2-TAP (kanMX) pep4∆::ADE2 sml1∆::HIS3 rad53∆::ADE2* |
| GDP1347 | This study | *MATa DPB2-TAP (kanMX) pep4∆::ADE2 sml1∆::HIS3 dun1∆::LEU2* |
| GDP1349 | This study | *MATa DPB2-TAP (kanMX) pep4∆::ADE2 mre11∆::kanMX* |
| GDP1351 | This study | *MATa DPB2-TAP (kanMX) pep4∆::ADE2 rad50∆::kanMX* |
| GDP1353 | This study | *MATa DPB2-TAP (kanMX) ctf18∆:: K.l. TRP1 pep4∆::ADE2* |
| GDP1355 | This study | *MATa DPB2-TAP (kanMX) mrc1∆:: K.l. TRP1 pep4∆::ADE2* |
| GDP1357 | This study | *MATa DPB2-TAP (kanMX) rad9∆::HIS3MX pep4∆::ADE2* |
| GDP1359 | This study | *MATa DPB2-TAP (kanMX) mrc1AQ-13MYC (his5+) pep4∆::ADE2* |
| GDP1380 | This study | *MATa DPB2-TAP (kanMX) SMC5-5FLAG (hphNT) sml1∆::HIS3 pep4∆::ADE2* |
| GDP1382 | This study | *MATa SMC5-5FLAG (hphNT) sml1∆::HIS3 pep4∆::ADE2* |
| GDP1386 | This study | *MATa MMS21-5FLAG (hphNT) sml1∆::HIS3 pep4∆::ADE2* |
| GDP1675 | This study | *MATa DPB2-TAP (kanMX) rrm3∆ (kanMX) sml1∆::HIS3 pep4∆::ADE2* |
| PJ69–4A | H. Ulrich | *MATa trp1-901 leu2-3,112 ura3-52 his3-200 gal4∆ gal80∆ LYS2::GAL1-HIS3 GAL2-ADE2 met2::GAL7-lacZ* |
| SS3 | K. Labib | *MATa pep4∆::ADE2* |
| yPZ604 | J Diffley | *MATa dbf4-4A (HIS3) sld3- 37A-10HIS-13MYC (KanMX)* |
