# Supplementary material for: The S phase checkpoint promotes the Smc5/6 complex dependent SUMOylation of Pol2, the catalytic subunit of DNA polymerase ε
Source: PLoS Genet. 2019 Nov 25;15(11):e1008427. doi: 10.1371/journal.pgen.1008427 (PMC6876773; doi:10.1371/journal.pgen.1008427)
Supplement: S2 Table — (DOCX) [file pgen.1008427.s010.docx]

Table S2 Plasmid list

| pCS206 | This study | pGBKT7- SMT3AA∆ |
| --- | --- | --- |
| pCS207 | This study | pGADT7-5GA-Pol2 (2013-2222) |
| pCS208 | This study | GADT7- Pol2 (2013-2192) |
| pCS209 | This study | pGADT7-Pol2 (2013-2222sim) F2210A I2212A L2213A L2214A |
| pCS210 | This study | pGADT7- Pol2 (2013-2222-CysA_mut) C2108S C2111S |
| pCS211 | This study | pGADT7- Pol2 (2013-2222-CysB_mut) C2164S C2167S |
| pHM27 | This study | pGADT7-5GA-Mrc1 |
| pKL273 | This study | pRS425 |
| pKL559 | Clonetech | pGADT7 (GAL4 AD) |
| pKL560 | Clonetech | pGBKT7 (GAL4 BD) |
| pKL1436 | Johnson, E.S | pET-UBC9-6HIS |
| pKL1437 | Johnson, E.S | pET-SMT3-6HIS |
| pKL1438 | Johnson, E.S | pET-Uba2/Aos1-6HIS |
| pKL1439 | Johnson, E.S | pET-Uba2/Aos1-6HIS |
| pKL1563 | This study | 5GA-SUMO(1-99) C-terminal tagging |
| pLG15 | K. Labib | pGADT7, POL2 (1-1265) |
| pLG17 | K. Labib | pGADT7, POL2 (1128-2222) |
| pLG18 | K. Labib | pGBKT7-POL2 (1128-2222) |
| pLG23 | This study | pGADT7-DPB2 |
| pLG24 | This study | pGBKT7-DPB2 |
| pLG47 | This study | pGADT7-5GA-Pol2 (1128-1483) |
| pLG48 | This study | pGADT7-5GA-Pol2 (1128-1777) |
| pLG49 | This study | pGADT7-5GA-Pol2 (1128-2107) |
| pLG69 | This study | pGADT7-5GA-Pol2 (1986-2222) |
| pLG99 | K. Labib | pGADT7-DPB3, 5GA linker |
| pLG100 | K. Labib | pGADT7-DPB4, 5GA linker |
| pLG119 | K. Labib | pGBKT7, DPB3, 5GA linker |
| pLG120 | K. Labib | pGBKT7, DPB4, 5GA linker |
